# Functional profiles of orphan membrane transporters in the life cycle of the malaria parasite

**DOI:** 10.1038/ncomms10519

**Published:** 2016-01-22

**Authors:** Sanketha Kenthirapalan, Andrew P. Waters, Kai Matuschewski, Taco W. A. Kooij

**Affiliations:** 1 Parasitology Unit, Max Planck Institute for Infection Biology, Charitéplatz 1, 10117 Berlin, Germany; 2 Wellcome Trust Centre for Molecular Parasitology, Glasgow Biomedical Research Centre, University of Glasgow, 120 University Place, Glasgow G12 8TA, UK; 3 Institute of Biology, Humboldt University, 10117 Berlin, Germany; 4 Department of Medical Microbiology, Radboud Institute for Molecular Life Sciences, Radboud University Medical Centre, PO Box 9101, 6500 HB Nijmegen, The Netherlands; 5 Centre for Molecular and Biomolecular Informatics, Radboud Institute for Molecular Life Sciences, Radboud University Medical Centre, PO Box 9101, 6500 HB Nijmegen, The Netherlands

## Abstract

Assigning function to orphan membrane transport proteins and prioritizing candidates for detailed biochemical characterization remain fundamental challenges and are particularly important for medically relevant pathogens, such as malaria parasites. Here we present a comprehensive genetic analysis of 35 orphan transport proteins of *Plasmodium berghei* during its life cycle in mice and *Anopheles* mosquitoes. Six genes, including four candidate aminophospholipid transporters, are refractory to gene deletion, indicative of essential functions. We generate and phenotypically characterize 29 mutant strains with deletions of individual transporter genes. Whereas seven genes appear to be dispensable under the experimental conditions tested, deletion of any of the 22 other genes leads to specific defects in life cycle progression *in vivo* and/or host transition. Our study provides growing support for a potential link between heavy metal homeostasis and host switching and reveals potential targets for rational design of new intervention strategies against malaria.

## Abstract (web summary)

The functions of many putative membrane transport proteins of malaria parasites are unknown. Here, Kenthirapalan *et al*. use mutant strains carrying targeted gene deletions to study the functions of 35 such proteins during the life cycle of *Plasmodium berghei* in mosquito and mouse hosts.

## Graphical abstract

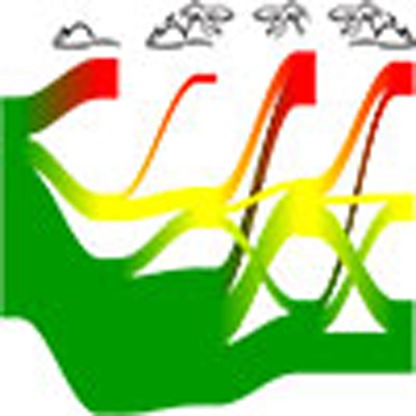

Membrane transport proteins (MTP) transfer compounds across biological membranes and encompass diverse gene families, namely ion channels, ATP-dependent pumps and secondary active porters including those of the major facilitator superfamily. Together they play important physiological roles in, for example, nutrient uptake, disposal of waste products, shuttling of metabolites between organelles, and generation and maintenance of the electrochemical gradient. They critically determine safety and efficacy of drugs and are attractive therapeutic targets^1^. Accordingly, MTPs rank amongst the top five protein classes that are molecular targets of FDA-approved drugs^2^. Prominent examples in the WHO model list of essential medicines include ion channel blockers, for example, verapamil, and serotonin transporter (5-HTT) inhibitors, for example, fluoxetine^3^.

In contrast to bacteria, archaea and fungi, parasitic protozoa such as *Trypanosoma brucei* and the malaria parasite *Plasmodium falciparum* allocate only a small proportion of their genomes (2–3%) to membrane transport (Supplementary Fig. 1)^4^. *P. falciparum* encodes at least 122 MTPs^5^. Some MTPs play central roles during the pathogenic blood-stage proliferation of malaria parasites, for example, through the import of critical nutrients such as pantothenic acid^6^,^7^ and isoleucine^8^, or mediate drug resistance, most notably against chloroquine through the chloroquine resistance transporter^9^,^10^. However, functions of the vast majority of *Plasmodium* transport proteins are inferred from homology to genes from model organisms^11^. For 39 gene products, functional or subcellular localization predictions remain elusive, rendering them orphan MTPs^5^. We reasoned that due to their phylogenetic distance to host MTPs they constitute particularly attractive targets for novel targeted malaria intervention approaches.

A better and unbiased understanding of human and pathogen *MTP* gene function is central to pharmacogenomics and drug target validation^12^. Despite this research priority, few systematic experimental genetics studies of MTPs have been reported for any organism and merely in the context of genome-wide collections of gene deletion mutants in model organisms, such as *Saccharomyces cerevisiae*^13^,^14^. In the search for targets for novel prophylactic, therapeutic and transmission-blocking intervention strategies to fight malaria, we report here a broad characterization of the importance of orphan MTP orthologues during the complete life cycle of the murine malaria model parasite *Plasmodium berghei* by relatively fast and efficient experimental genetics approaches.

## Results and Discussion

### Enrichment of putative flippases in vital gene candidates

**Figure 1:**
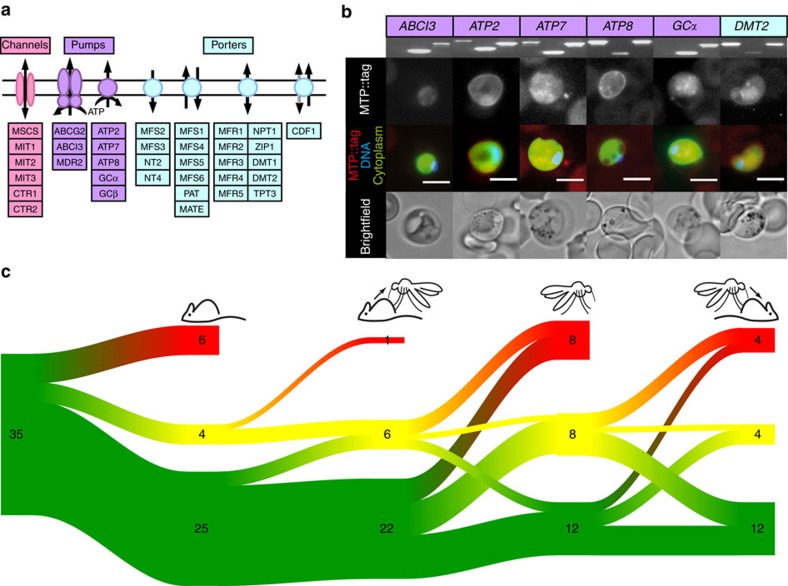
Experimental genetics screen of malaria parasite orphan membrane transport proteins. (**a**) Schematic overview of the 35 conserved, *P. berghei* orphan MTPs targeted in this study. (**b**) Endogenous fluorescent tagging of six MTPs refractory to targeted gene deletion to validate accessibility of gene loci. Diagnostic PCRs of the parental parasite populations (left, WT; centre, 5′ integration; right, 3′ integration; see Supplementary Fig. 5 for full gel pictures) and representative live fluorescent micrographs of blood-stage parasites are shown. Scale bars, 5 μm. (**c**) Sankey diagram for parasite development at four life cycle checkpoints. Parasite lines are enumerated and coloured according to complete arrest (red), slow development (<10% percentile; yellow), and normal growth (green). The Sankey diagram was created with the package riverplot (v. 0.5) available from CRAN (https://cran.r-project.org/web/packages/riverplot/).

**Figure 2:**
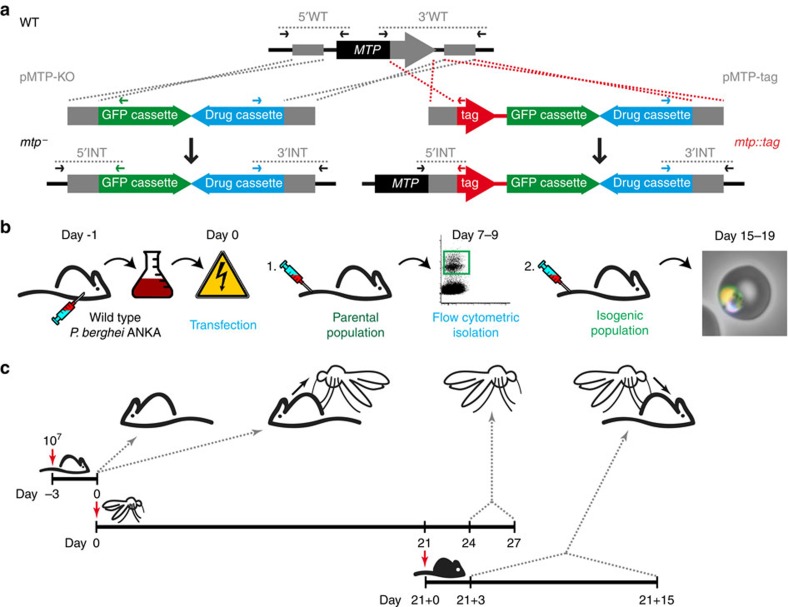
Experimental genetics approaches employed to study 35 MTP. (**a**) Schematic representation of the generation of *mtp*^−^ and *mtp::tag* parasites. The 5′ and 3′ flanking regions of the target genes were cloned adjacent to the selection cassette resulting in the gene deletion transfection vector (pMTP-KO). For endogenous tagging, the carboxy terminus was cloned in frame with an mCherry-3xMyc tag and the 3′FR was cloned distal of the selection cassette, resulting in the endogenous tagging vector (pMTP-tag). By double crossover homologous recombination the targeted MTP was predicted to be either replaced or endogenously tagged with the fluorescent tag, respectively. (**b**) Schematic representation of the *P. berghei* transfection protocol adapted from Matz and Kooij^37^. Cultured and synchronized schizonts are transfected and successfully modified parasites are selected *in vivo* using pyrimethamine. When the parasitaemia is 0.1–1.0%, 50 isogenic mutant parasites are isolated by flow cytometry. (**c**) Schematic overview of the standardized phenotypic profiling protocol of four life cycle checkpoints looking at (1) blood-stage growth in the mouse, (2) exflagellation rates as a measure of mouse-to-mosquito transmission, (3) salivary gland sporozoite numbers, and (4) prepatency following infections by natural bites to follow mosquito-to-mouse transition and ability to complete the full life cycle.

**Figure 3:**
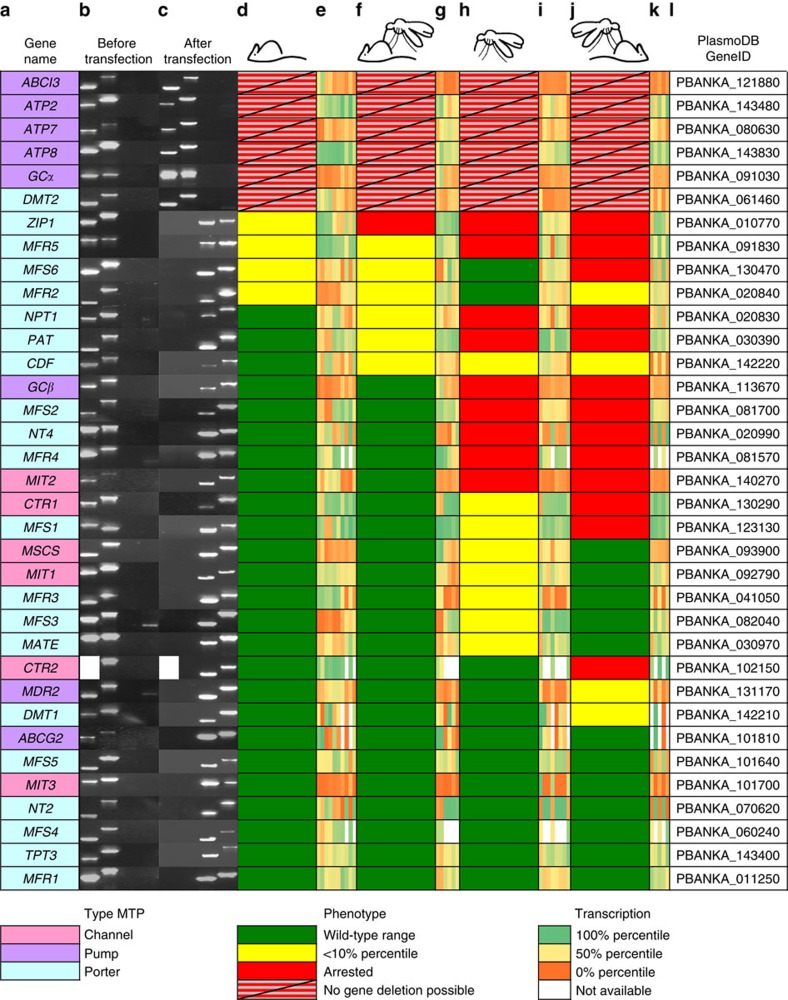
Genetic screen of 35 *Plasmodium berghei* membrane transport proteins. The 6 essential and 29 targetable genes were clustered according to their phenotypes. For each gene the following information is provided: (**a**) gene name and MTP category colour-coded as indicated in the legend; (**b**) diagnostic PCR before transfection on DNA of WT parasites specific for 5′ and 3′ WT, 5′ and 3′ integration (note that for WT *CTR2* a single overarching PCR from 5′ to 3′ was performed; see Supplementary Fig. 5 for full gel pictures); (**c**) diagnostic PCR after transfection on DNA of parental transfected populations (6 essential genes) or isolated, isogenic mutant parasites (29 targetable genes) specific for 5′ and 3′ WT, 5′ and 3′ integration—absence of WT signals indicate 29 pure populations (see Supplementary Fig. 5 for full gel pictures); (**d**–**k**) alternating graphic representations of the phenotypes and transcription levels derived from refs 38, 39, 40, colour coded as indicated in the legend; (**d**) blood-stage growth measured as pparasitaemia 3 days after infection of NMRI mice with 10^7^ parasites; (**e**) transcription percentiles of *P. berghei* ring stages 4 hours post infection (h.p.i.; 2 ×), trophozoites 16 h.p.i. (2 ×), schizonts 22 h.p.i. (2 ×), *P. yoelii* schizonts (2 ×), and mixed blood stages (2 ×); (**f**) male gamete exflagellation levels three days after infection of NMRI mice with 10^7^ parasites; (**g**) transcription percentiles of *P. berghei* gametocytes (2 ×) and *P. yoelii* gametocytes (4 ×); (**h**) number of salivary gland-associated sporozoites 21 days after blood meal; (**i**) transcription percentiles of *P. berghei* ookinetes, *P. yoelii* midgut-associated sporozoites 9 d.p.i. (2 × ), 10 d.p.i., salivary gland-associated sporozoites 14 d.p.i. (3 × ) and 15 d.p.i.; (**j**) prepatent period in two C57BL/6 mice following bites of 25 infectious mosquitoes (green, both mice became blood-film positive with an average prepatent period of ≤4.5 days; yellow, one mouse remained blood-film negative; red, both mice remained blood-film negative); (**k**) transcription percentiles of *P. yoelii* liver stages at 24, 36, 40 (2 × ) and 50 h.p.i.; (**l**) gene identification number.

For three of the 39 *P. falciparum* orphan MTPs there is no rodent malaria parasite orthologue (Fig. 1a; Supplementary Table 1). In addition, *GAP40* encodes a member of the glideosome motor complex^15^. As predicted, *PbGAP40* is refractory to constitutive gene deletion (Supplementary Fig. 2). Of the remaining 35 *P. berghei* orphan MTPs, only six (17%) were refractory to repeated gene deletion attempts, using two complementary strategies (Fig. 2a,b)^16^,^17^, strongly indicating essential roles during asexual blood-stage growth (Figs 1b,c and 3). Corresponding gene deletion lines (*mtp*^−^) could not be generated, but we readily selected endogenously labelled *mtp::tag* lines (Fig. 1b). Live fluorescent imaging of intra-erythrocytic parasites revealed localization at the parasite–host interface (ATP2 and ATP8) or to intraparasitic structures and the surrounding membranes (ABCI3, ATP7, GCα and DMT2). Intriguingly, four essential genes encode signatures of aminophospholipid-transporting P_4_-type ATPases. These ATPases are restricted to eukaryotes and facilitate inward translocation of aminophospholipids thereby maintaining their asymmetrical enrichment at the membrane inner leaflet^18^. As lipid asymmetry is critical to normal cell functions, our data are consistent with a vital dependence of blood-stage malaria parasites on maintenance of lipid asymmetry. This potential vulnerability was previously unrecognized and might inform drug discovery programs.

### Streamlined phenotyping of viable *mtp* ^−^ lines

For 29 target genes (81%), transfection, selection and isolation of isogenic *mtp*^–^ lines were successful (Figs 1, 2, 3). We established a streamlined and standardized phenotypic screen (Fig. 2c), monitoring life cycle progression at four clearly defined checkpoints in the definitive host, female *Anopheles* mosquitoes, and the intermediate murine host. Following intravenous infection of outbred (NMRI) mice with 10^7^ infected erythrocytes, parasitaemia (i) and male gamete exflagellation (ii) were quantified three days later. *Anopheles stephensi* mosquitoes were allowed to feed on these mice and salivary gland-associated sporozoites (iii) were enumerated at least three weeks later. Natural transmission (iv) was monitored after exposure of C57BL/6 mice to 25 *mtp*^−^-infected mosquitoes.

This screen identified only seven gene deletions with no apparent deficiency at any checkpoint, indicative of dispensable and/or redundant roles for parasite propagation and host switch (Figs 1c and 3; Supplementary Table 2). Ablation of nine *MTP* genes resulted in defects at a single stage or at multiple checkpoints. Despite the observed deficiencies, these *mtp*^−^ lines completed the life cycle, illustrating that life cycle bottlenecks can be readily compensated by subsequent propagation phases in partly attenuated parasites. A selection of seven mutants exemplified how such an initial defect does not need to preclude subsequent normal development. Sporozoite numbers for the *mfs6*^−^ and *mfr2*^−^ strains are within the wild-type (WT) range despite severely reduced exflagellation rates, while five mutant lines that show reduced sporozoite numbers transmit normally. The remaining thirteen *mtp*^−^ lines (36%) demonstrated a complete life cycle arrest abrogating transmission between mice.

### Defects during asexual or sexual blood-stage development

**Figure 4:**
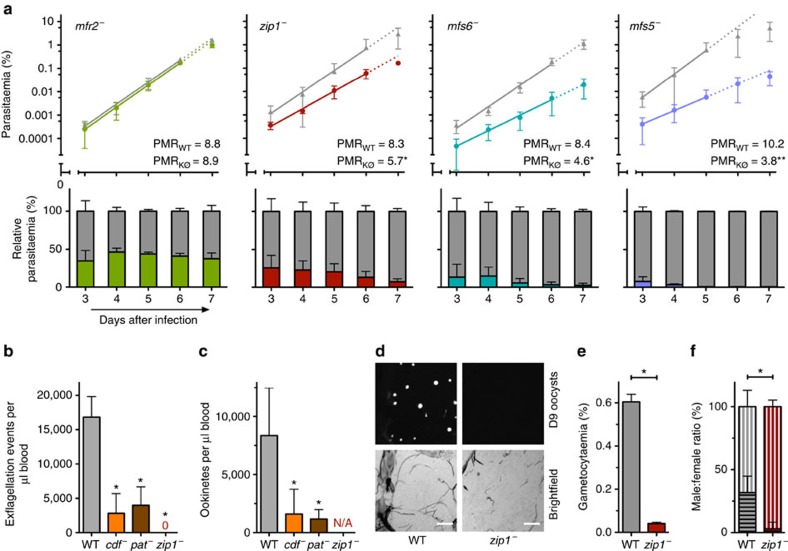
Blood infection and mouse-to-mosquito transition of selected *mtp*^−^ parasites. (**a**) Blood infection of the four slowest growing *mtp*^−^ parasite lines (*mfr2*^−^, *zip1*^−^, *mfs6*^−^, and *mfr5*^−^) in competition with WT (grey). Shown are mean parasitaemias±s.d. from at least three experiments (top) and the kinetics of relative parasite distribution (bottom) from day 3 to 7 after injection. **P<0.01; *P<0.05 (two-tailed F-test). (**b**) Male gamete exflagellation and (**c**) *in vitro* ookinete formation for selected *mtp*^−^ (*cdf*^−^, *pat*^−^ and *zip1*^−^) parasite lines. Shown are mean values±s.d. *P<0.05 (Mann–Whitney test). (**d**) Oocysts formation is abrogated in *zip1*^−^ parasites. Bars, 100 μm. (**e**) Gametocyte conversion is defective in *zip1*^−^ parasites. Shown are mean values±s.d. *P<0.05 (Mann–Whitney test). (**f**) Ratio of male (bottom, horizontal lines) and female (top, vertical lines) gametocytes of the total formed in WT and *zip1*^−^ infections shows that male gametocyte formation in *zip1*^−^ parasites is more defective than female gametocyte formation. Shown are mean values±s.d. *P<0.05 (Mann–Whitney test).

We next determined the parasite multiplication rates (PMR) of the four parasite lines that showed the lowest mean parasitaemias in the phenotyping screen (Fig. 4a). Thus, we could rule out a growth defect for *mfr2*^−^ but uncovered replication defects for *zip1*^−^ (68% PMR), *mfs6*^−^ (55% PMR) and *mfr5*^−^ (36% PMR) parasites, resulting in swift out-competition by WT parasites. Growing at a third of the rate of WT blood-stage parasites, *MFR5*-deficient parasites are the slowest replicating mutants isolated to date and we independently confirmed this outcome (Supplementary Fig. 3).

We detected substantial reductions in male gamete exflagellation and ookinete formation for *pat*^−^ and *cdf*^−^ (Fig. 4b,c). As *cdf*^−^, but not *pat*^−^, parasites were capable of completing transmission (Fig. 3; Supplementary Table 2) *PAT* likely exerts additional roles following ookinete formation. Remarkably, *zip1*^−^ was the only mutant that completely failed to form flagella and, thus, ookinetes and oocysts (Fig. 4b–d). In this mutant, gametocyte numbers were reduced by 80% (Fig. 4e) but most strikingly the male:female ratio was strongly skewed towards female production (Fig. 4f). *CDF* and *ZIP1* might participate in the transport of heavy metal ions^5^,^19^. Our phenotyping, and the known role of a copper-transporting P_1B1_-type ATPase (CuTP) in gamete fertility^20^, underscore a possible central role for heavy metal homeostasis in malaria parasites fertility and, perhaps, the germ line of other eukaryotes.

### Defects during mosquito-stage development

**Figure 5:**
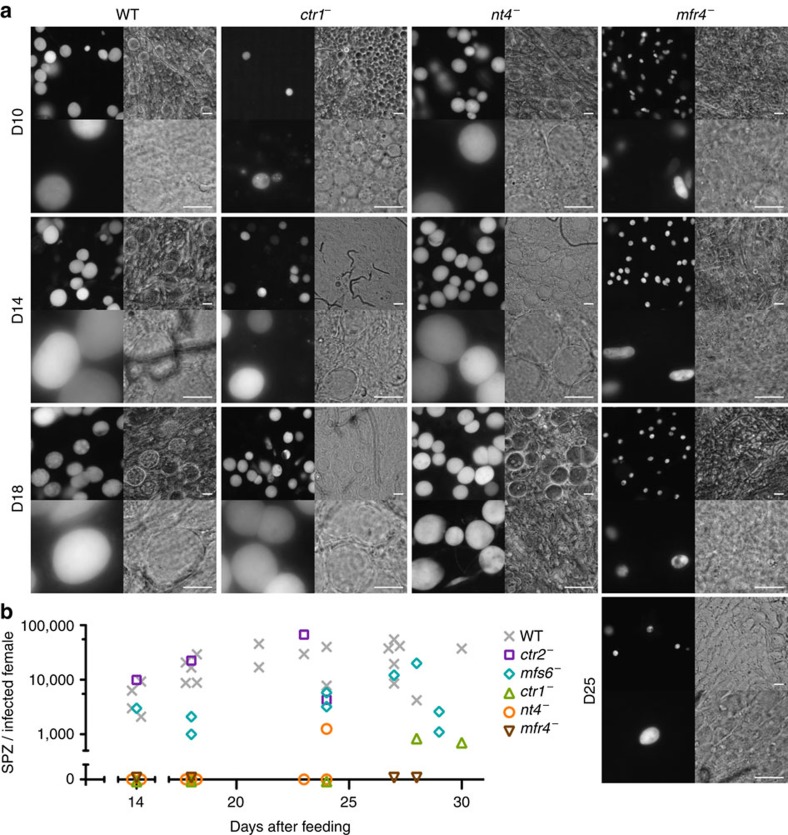
Critical roles for selected *MTPs* in sporogony. (**a**) Mosquito midguts were isolated at 10, 14, 18 and 25 (*mfr4*^−^ only) days after the infectious blood meal. Shown are representative live micrographs of the highly expressed cytoplasmic GFP (left panels) and bright field images (right panels) recorded with 40 × (top panels) and 100 × (bottom panels) objectives. Note the elongated ookinete-like shape rather than a round and spherical appearance of the *mfr4*^−^ oocysts in the higher magnification. Scale bars, 20 μm. (**b**) Enumeration of sporogony in five recombinant parasite lines. Midgut-associated sporozoites were isolated and quantified on day 14 after the blood meal. Salivary gland-associated sporozoite numbers were determined over the course of two weeks starting on day 18 after the infectious blood meal. Data combined from at least three independent feeding experiments.

Over one third of the targeted *MTP* genes displayed a first defect in sporozoite colonization of the salivary glands (Figs 1c and 3, and Supplementary Table 2). These included four of six predicted channels (*CTR1*, *MIT1*, *MIT2* and *MSCS*), highlighting their importance for the extracellular growth stage of the parasite. Five *mtp*^−^ lines did not produce any salivary gland-associated sporozoites and two mutants failed to transmit following strongly reduced sporozoite production. In-depth phenotyping of three representative mutants (*ctr1*^−^, *nt4*^−^ and *mfr4*^−^) showed normal midgut colonization but attenuation of oocyst development, resulting in either no sporozoites (*mfr4*^−^) or severely reduced sporozoite colonization of salivary glands (*ctr1*^−^ and *nt4*^−^; Fig. 5).

### Defects during liver-stage development

**Figure 6:**
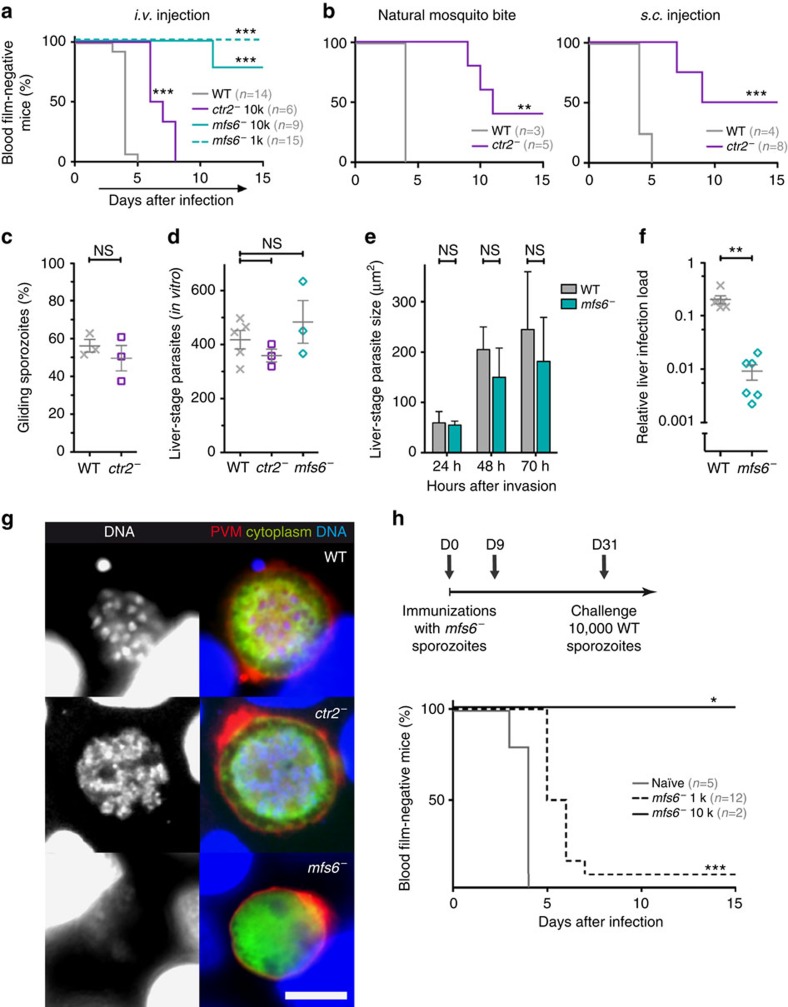
Defective transmission and liver-stage maturation in *ctr2*^−^ and *mfs6*^−^ parasites. (**a**) Kaplan–Meier analysis of infection by intravenous injection of 10,000 (WT *n*=14, *ctr2*^−^ *n*=6, *mfs6*^−^ *n*=9) or 1,000 (*mfs6*^−^ *n*=15) salivary gland-associated sporozoites. ***P<0.001 (Log-rank Mantel–Cox). (**b**) Kaplan–Meier analysis of infection by exposure to infected mosquitoes (left; WT *n*=3, *ctr2*^−^ *n*=5) and subcutaneous injection of 10,000 isolated salivary gland-associated sporozoites (right; WT *n*=4, *ctr2*^−^ *n*=8). ***P<0.001; **P<0.01 (Log-rank Mantel–Cox). (**c**) Gliding motility of *ctr2*^−^ sporozoites (NS, non-significant; Mann–Whitney test). (**d**) Numbers of mature *ctr2*^−^ and *mfs6*^−^ liver stages in cultured hepatoma cells (NS, non-significant; Kruskal–Wallis with Dunn's post test). (**e**) Surface areas of developing *mfs6*^−^ liver-stage parasites. Shown are mean values±s.d. (NS, non-significant; Mann–Whitney test). (**f**) Parasite liver load in WT- and *mfs6*^−^-infected C57BL/6 mice. **P<0.01 (two-tailed Mann–Whitney). (**g**) Aberrant liver-stage maturation of *mfs6*^−^ parasites. Shown are immunofluorescent micrographs of mature liver-stage-infected hepatoma cells. (UIS4, red; HSP70, green; Hoechst, blue). Scale bar, 10 μm. (**h**) Immunization/challenge protocol (top) for vaccination with genetically arrested *mfs6*^−^ sporozoites. Kaplan–Meier analysis (bottom) of blood infection after sporozoite challenge infection in *mfs6*^−^-immunized mice. ***P<0.001; *P<0.05 (Log-rank Mantel–Cox).

Only two mutants with normal sporozoite production, *ctr2*^−^ and *mfs6*^−^, had a distinct defect during natural transmission (Figs 3 and 5b; Supplementary Table 2). The observed defect in natural transmission of *ctr2*^−^ parasites (Fig. 6a) was alleviated by intravenous, but not subcutaneous, syringe delivery of sporozoites (Fig. 6b), and was largely independent of gliding motility (Fig. 6c) or liver-stage maturation (Fig. 6d,g). These findings indicate an important role for *CTR2* in sporozoite transmission *in vivo*. The critical roles of an alternative zinc–iron permease (*ZIPCO*) during liver-stage development^21^, and of *CTR1* and *CTR2* described herein, support a link between heavy metal homeostasis and parasite–host switch. Furthermore, iron deprivation via hepcidin inhibits liver-stage growth^22^. Taken together, the growing evidence indicates that heavy metal homeostasis might become a useful molecular target for causal prophylactic strategies.

Genetically modified *Plasmodium* parasites that are unable to undergo liver-to-blood-stage conversion can be used to experimentally immunize mice resulting in sterile protection against future sporozoite challenge infections^23^. Broadly, these genetically arrested parasites fall into two distinct classes: (1) early arrested liver stages that fail to grow in cultured hepatoma cells and show a reduced liver load *in vivo*, and (2) late arrested parasites that initially grow normally but have a defect in liver-stage merozoite formation, correlating with a high liver load in infected mice. Only one of the mutant strains, *mfs6*^−^, fulfilled criteria for testing in a preclinical immunization/challenge protocol as a genetically arrested parasite vaccine. Fine analysis of the observed defect of *mfs6*^−^ parasites in natural transmission revealed occasional (2 out of 9) delayed breakthrough blood infections only after high-dose inoculations with 10,000 *mfs6*^−^ sporozoites (Fig. 6a). Numbers and sizes of liver-stage parasites in cultured hepatoma cells were not different from WT parasites (Fig. 6d,e), though a trend towards slightly reduced sizes in maturing stages was observed. Furthermore, we did not detect liver-stage merozoites in *mfs6*^−^-infected cell cultures. In contrast with late arresting liver-stage mutants, *mfs6*^−^ liver load was largely reduced *in vivo* (Fig. 6f). Intriguingly, DNA staining revealed distorted nuclei displaying weak signals only (Fig. 6g; Supplementary Fig. 4), lending support to an apparent replication deficit. Therefore, infections with *mfs6*^−^ parasites display defining signatures of late arrest (numbers and morphology) and early arrest (DNA content).

Immunizations with late arrested parasites elicit particularly potent and lasting protection^24^,^25^. To test the vaccine potential of *mfs6*^−^ parasites, we immunized C57BL/6 mice with two low doses of 1,000 *mfs6*^−^ sporozoites. The immunized mice displayed an average delay to blood infection of 1–2 days upon high-dose sporozoite challenge and one mouse was sterily protected from re-infection (Fig. 6h). Despite this promising result, additional bioengineering efforts, including generation of multiple gene deletions, are required before a late arresting genetically attenuated whole-parasite vaccine can be translated to *P. falciparum* parasites and potential clinical trials.

### Concluding remarks

Classical reverse genetics and post-genomic approaches continue to provide increasing insights in the functioning of membrane transport proteins in the model yeast *S. cerevisiae*^26^. In the present study, we have presented a relatively rapid experimental system to uncover phenotypes and assign *in vivo* functions to a significant proportion (30%) of the *Plasmodium* transporters, which could not be inferred on the basis of motifs and sequence similarities with established unicellular model organisms. Such insights contribute to a better understanding of *Plasmodium* metabolism and host cell adaptation, which forms the basis for the rational development of innovative malaria intervention strategies. Our data show that MTPs, with the notable exception of ATP-dependent pumps, play a less prominent role than anticipated. Systematic target validation is a critical component of the anti-malarial drug and vaccine discovery process. This is exemplified herein by the prophylactic and transmission-blocking potential of targeting an MTP putatively involved in heavy metal homeostasis during malaria parasite–host switches; the vaccine potential of genetically arrested parasites that lack a parasite-specific MTP of the major facilitator superfamily; and the possibility for therapeutic intervention targeting putative aminophospholipid translocases.

## Methods

### Experimental animals

All animal work was conducted in accordance with the German ‘Tierschutzgesetz in der Fassung vom 18. Mai 2006 (BGBl. I S. 1207)', which implements the directive 86/609/EEC from the European Union and the European Convention for the protection of vertebrate animals used for experimental and other scientific purposes. The protocol was approved by the ethics committee of MPI-IB and the Berlin state authorities (LAGeSo Reg# G0469/09). We used 6-to-8 weeks old female mice from Charles River: C57BL/6 mice for sporozoite infections and NMRI mice for all other parasite infections.

### Generation of *mtp* ^−^ and *mtp::tag* parasites

All recombinant parasite lines were generated in the *P. berghei* strain ANKA reference line cl15cy1 (ref. 27) through a gene replacement strategy via double crossover/ends-out homologous recombination (Fig. 2a). All sequences of the oligonucleotides with the restriction sites and approximate sizes can be found in Supplementary Table 3. Fragments of 400–650 bp in the 3′ flanking regions of the genes of interest were amplified by using the 3′ fragment forward and reverse primer combinations (3′MTP-F-AvrII and 3′MTP-R-KpnI). The 3′ fragments were cloned using the indicated restriction sites into the pBAT-SIL6 vector^28^, resulting in intermediate vectors (pMTP-IM). For completion of the gene deletion vectors (pMTP-KO), 400–650 bp fragments in the 5′ flanking regions of the target genes were amplified using the 5′ fragment forward and reverse primer combinations (5′MTP-F-SacII and 5′MTP-R-HpaI) and cloned into the pMTP-IM vectors from which the mCherry-3xMyc tag sequence was removed by restriction digestion with SacII and PvuII. For completion of the tagging vectors (pMTP-tag), 400–650 bp fragments at the carboxy-terminal (CT) ends of the coding sequences were amplified using the CT fragment forward and reverse primer combinations (CT-MTP-F-SacII and CT-MTP-R-HpaI) and cloned into the pMTP-IM vectors using SacII and HpaI resulting in in-frame fusions of the CT coding regions with the mCherry-3xMyc tag. All transfection plasmids were verified by commercial Sanger sequencing and linearized with the enzymes ScaI and SalI.

We transfected *P. berghei* strain ANKA with 5 μg linearized transfection plasmid DNA using standard procedures (Fig. 2b)^27^. In brief, blood of a mouse infected with WT parasites is harvested by cardiac puncture and cultured overnight. Following maturation, the parasites fail to egress and arrest at the schizont stage. These schizonts are purified and transfected with the targeting constructs. Transfected merozoites are injected intravenously into a naïve mouse. Administration of pyrimethamine in the drinking water favours the growth of successfully modified parasites, which now also express a fluorescent protein. When the parasitaemia is 0.1–1.0% (typically 7–9 days after transfection), 50 mutant parasites are isolated by flow cytometry and transferred to a naïve mouse^17^. Eight to ten days after injection, the isogenic parasite line can be harvested, stored or transferred and tested.

When three repeated gene deletion attempts were unsuccessful, refractoriness of the genes was confirmed using the available, corresponding *Plasmo*GEM vectors (*GAP40*, PbG01-2401a08; *ABCI3*, PbG01-2370b05; *ATP2*, PbG01-2339e11; *GCα*, PbG01-2474g01)^16^. These independent transfection vectors harbour significantly longer homology arms and integrate more efficiently providing a further, more stringent test for essentiality. Accessibility of the locus for genetic manipulation was confirmed using the pMTP-tag vectors.

Correct integration of the transfection vectors in the isogenic parasite lines and absence of contaminating WT parasites was verified by genotyping PCR (Figs 2a and 3; Supplementary Table 3). In general, for each target gene two primer combinations for genotyping purposes were designed just outside the regions used for homologous recombination, one flanking the 5′ fragment (5′MTP-F and 5′MTP-R) and one spanning both the CT- and 3′ fragments (3′MTP-F and 3′MTP-R). To confirm the predicted integration events, the gene-specific primers were combined with generic pBAT-SIL6-specific primers (5′HSP70rev for 5′ integration in *mtp*^−^ lines, mCherryRev for 5′ integration in *mtp::tag* lines, and 5′DHFRrev for 3′ integration in both).

### Standardized phenotyping screen

To facilitate a comprehensive phenotypic profiling of the large number of recombinant *mtp*^−^ parasite lines, we standardized our life cycle progression analysis to maximize functional read-outs over invested time and resources, particularly with regard to the number of experimental animals required. Figure 2c provides a schematic of this protocol, which starts with the intravenous injection of 10^7^ blood-stage parasites into naïve NMRI mice. Three days later, blood-stage parasitaemia was counted by microscopic examination of Giemsa-stained thin blood films and male gamete exflagellation rates were determined as described below. For transmission, 25 female naïve *A. stephensi* Sind-Kasur strain mosquitoes^29^ were placed into a single cup, starved for at least 8 h, and fed on a *mtp*^−^-infected mouse for 30 min. After 21 days, infected mosquitoes were starved for at least 8 h and fed on naïve C57BL/6 mice. From 3 to 15 days after the infectious mosquito bites, blood-stage infection was monitored by microscopic examination of Giemsa-stained thin blood films. At 24–27 days after the mosquito blood meal, salivary gland-associated sporozoites were isolated and quantified.

### Blood-stage development and live fluorescence imaging

Blood-stage development of selected *mtp*^−^ lines was analysed using a flow cytometry-based intravital competition assay, growing recombinant parasite lines in competition with a strongly fluorescent reference strain (Beryellow or Berred) within a single mouse^30^. Data from the exponential growth phase, that is, with parasitaemia <1%, fitted a linear regression well (*r*^2^≥0.99) and allowed the calculation of the PMR from the slopes. For live protein localization, a drop of tail blood from an infected mouse was mixed with 200 μl pre-warmed RPMI 1640 complemented with 0.2 μl of the DNA-dye Hoechst 33342 (Invitrogen) and distributed onto poly-L-lysine coated cover slips. Cells were allowed to settle for 5 min at 37 °C. Next, cover slips were washed three times with pre-warmed RPMI, inverted and sealed. Images were recorded on a Zeiss AxioObserver Z1 epifluorescence microscope, equipped with a Zeiss AxioCam MRm camera, and processed minimally with Fiji^31^.

### Sexual blood-stage development and ookinete cultures

Detailed analyses of sexual development was largely performed as described^20^. Mice were infected with 10^7^ blood-stage parasites. After 3 days, gametocyte conversion rates and male:female gametocyte ratios were determined by microscopic examination of Giemsa-stained thin blood films. For exflagellation analysis, 5 μl of tail blood was mixed immediately with 125 μl RPMI 1640 complemented with 50 μM xanthurenic acid, that was pre-warmed at 20 °C. Immediately, 10 μl cell suspension was transferred into a Neubauer chamber and incubated at 20 °C. Exflagellation centres were quantified by microscopic observation at 400 × magnification from 12 to 18 min. Finally, mice were bled by heart puncture and 1 ml blood was transferred immediately into a small cell culture flask containing 10 ml complete ookinete medium (RPMI 1640 with L-glutamine and 25 mM HEPES supplemented with 100 mM sodium bicarbonate, 125 U ml^−1^ penicillin/streptomycin, 10% fetal calf serum and 50 μM xanthurenic acid, pH 8.0). After 18–24 h incubation at 20 °C and 80% humidity, the ookinete culture was centrifuged in a 50 ml reaction tube for 8 min at 1,800 r.p.m. (560*g*) without brake. The pellet was resuspended with freshly prepared cold 0.17 M ammonium chloride solution and incubated for 10–15 min on ice. Ookinetes were collected by centrifugation at 4 °C and 2,100 r.p.m. (760*g*) for 15 min. The pellet was transferred into a 2 ml reaction tube and washed twice with PBS. The enriched ookinete suspension was then diluted appropriately, 10 μl of it transferred into a Neubauer chamber and quantified by microscopic examination at 400 × magnification.

### Mosquito-stage development and live fluorescence imaging

*A. stephensi* mosquitoes were raised in a 14 h light/10 h dark cycle at 75% humidity using standard techniques^32^. Uninfected and infected mosquitoes were kept at 28 and 20 °C, respectively. Midgut sporozoites were isolated and quantified 14 days after the mosquito blood meal. Salivary gland-associated sporozoites were isolated and quantified between day 18 and 30 following the start of the mosquito infection. To visualize mosquito-stage development, midguts were isolated in RPMI 1640 supplemented with 2% BSA at 10, 14, 18 and 25 days after the mosquito blood meal. Mosquito midguts were placed on a glass slide and images recorded immediately using a Leica DMR epifluorescence microscope with 40 × and 100 × objectives.

### Gliding motility assay

Glass slides with distinct wells were coated with 3% BSA in RPMI at 37 °C for 20 min. Overall 10,000 sporozoites in RPMI/3% BSA were added onto each well. Samples were placed at 37 °C in a humid chamber to allow sporozoites to settle and glide for 45 min. The parasites were fixed with 4% PFA for 15 min. Parasites and their trails were stained with anti-CSP antiserum (1:500 dilution—kindly provided by K. Müller, MPI-IB, Berlin). Primary antibodies were detected by fluorescently labelled secondary goat anti-mouse IgG Alexa Fluor 488 conjugated antibodies (1:1,000 dilution, Invitrogen). Sporozoites with trails that extended longer than one circle were scored as gliding. Data were obtained in three independent assays using salivary gland-associated sporozoites isolated from mosquitoes that were fed simultaneously.

### Transmissions to mice and quantification of liver loads

For natural transmission, infected mosquitoes were starved for at least 8 h before feeding on anesthetized naïve C57BL/6 mice. For intravenous and subcutaneous injections in C57BL/6 mice, the desired numbers of sporozoites, typically 10,000, were isolated from infected mosquito salivary glands and injected in a volume of 100 μl into the tail vein or a skin fold on the back. To quantify the relative parasite liver infection load, 10,000 sporozoites were injected intravenously into C57BL/6 mice. After 44 h, the mice were sacrificed, RNA was isolated from infected livers, and transcribed into cDNA as described previously^33^. Quantitative real time PCR was performed on WT and mutant cDNA by using primers specific for *P. berghei 18S* rRNA and mouse glyceraldehyde-3-phosphate dehydrogenase (*GAPDH*)^33^. The mRNA level of *P. berghei 18S* rRNA was calculated relative to the mouse *GAPDH* levels.

### *In vitro* liver-stage growth and immunofluorescence imaging

To study liver-stage development, *in vitro* cultured human hepatoma (Huh7) cells^34^ were infected with 10,000 sporozoites using standard techniques as described previously^25^. After 24, 48 or 70 h, liver-stage parasites were fixed and permeabilized with ice-cold methanol and stained with the DNA-dye Hoechst 33342 (Invitrogen), rabbit anti-UIS4 antibodies (1:500 dilution^35^), and mouse anti-HSP70 antibodies (1:300 dilution^36^). Primary antibodies were detected by fluorescently labelled goat anti-mouse/rabbit IgG Alexa Fluor 488/546 conjugated antibodies (1:1,000 dilution, Invitrogen). Liver-stage parasites were counted and images recorded on a Zeiss AxioObserver Z1 epifluorescence microscope, equipped with a Zeiss AxioCam MRm camera, and processed minimally with Fiji^31^.

### Immunizations with *mfs6* ^−^ sporozoites

C57BL/6 mice were immunized with two doses of 1,000 or 10,000 *mfs6*^−^ sporozoites by intravenous injection at a 9-day interval. Mice that remained parasite-free were challenged three weeks after the final immunization with 10,000 WT sporozoites. Blood-stage infection was monitored daily from days 3–15 after infection by microscopic examination of Giemsa-stained thin blood films.

## Additional information

**How to cite this article:** Kenthirapalan, S. *et al*. Functional profiles of orphan membrane transporters in the life cycle of the malaria parasite. *Nat. Commun.* 7:10519 doi: 10.1038/ncomms10519 (2016).

## Supplementary Material

Supplementary Information Supplementary Figures 1-5, Supplementary Tables 1-3 and Supplementary References

## References

[b1] International Transporter Consortium. . Membrane transporters in drug development. Nat. Rev. Drug. Discov. 9, 215–236 (2010). 20190787 10.1038/nrd3028 PMC3326076

[b2] Overington J. P., Al-Lazikani B. & Hopkins A. L. How many drug targets are there? Nat. Rev. Drug. Discov. 5, 993–996 (2006). 17139284 10.1038/nrd2199

[b3] World Health Organization. WHO model list of essential medicines. 1–55 (2015). http://www.who.int/medicines/publications/essentialmedicines/en/.

[b4] Ren Q., Chen K. & Paulsen I. T. TransportDB: a comprehensive database resource for cytoplasmic membrane transport systems and outer membrane channels. Nucleic Acids Res. 35, D274–D279 (2007). 17135193 10.1093/nar/gkl925 PMC1747178

[b5] Martin R. E., Ginsburg H. & Kirk K. Membrane transport proteins of the malaria parasite. Mol. Microbiol. 74, 519–528 (2009). 19796339 10.1111/j.1365-2958.2009.06863.x

[b6] Saliba K. J., Horner H. A. & Kirk K. Transport and metabolism of the essential vitamin pantothenic acid in human erythrocytes infected with the malaria parasite *Plasmodium falciparum*. J. Biol. Chem. 273, 10190–10195 (1998). 9553068 10.1074/jbc.273.17.10190

[b7] Augagneur Y. . Identification and functional analysis of the primary pantothenate transporter, *Pf*PAT, of the human malaria parasite *Plasmodium falciparum*. J. Biol. Chem. 288, 20558–20567 (2013). 23729665 10.1074/jbc.M113.482992 PMC3711320

[b8] Martin R. E. & Kirk K. Transport of the essential nutrient isoleucine in human erythrocytes infected with the malaria parasite *Plasmodium falciparum*. Blood 109, 2217–2224 (2007). 17047158 10.1182/blood-2005-11-026963

[b9] Fidock D. A. . Mutations in the *P. falciparum* digestive vacuole transmembrane protein *Pf*CRT and evidence for their role in chloroquine resistance. Mol. Cell 6, 861–871 (2000). 11090624 10.1016/s1097-2765(05)00077-8 PMC2944663

[b10] Martin R. E. . Chloroquine transport via the malaria parasite's chloroquine resistance transporter. Science 325, 1680–1682 (2009). 19779197 10.1126/science.1175667

[b11] Martin R. E., Henry R. I., Abbey J. L., Clements J. D. & Kirk K. The ‘permeome' of the malaria parasite: an overview of the membrane transport proteins of *Plasmodium falciparum*. Genome Biol. 6, R26 (2005). 15774027 10.1186/gb-2005-6-3-r26 PMC1088945

[b12] Evans W. E. & Relling M. V. Pharmacogenomics: translating functional genomics into rational therapeutics. Science 286, 487–491 (1999). 10521338 10.1126/science.286.5439.487

[b13] Winzeler E. A. . Functional characterization of the *S. cerevisiae* genome by gene deletion and parallel analysis. Science 285, 901–906 (1999). 10436161 10.1126/science.285.5429.901

[b14] Giaever G. . Functional profiling of the *Saccharomyces cerevisiae* genome. Nature 418, 387–391 (2002). 12140549 10.1038/nature00935

[b15] Frénal K. . Functional dissection of the apicomplexan glideosome molecular architecture. Cell Host. Microbe 8, 343–357 (2010). 20951968 10.1016/j.chom.2010.09.002

[b16] Pfander C. . A scalable pipeline for highly effective genetic modification of a malaria parasite. Nat. Methods 8, 1078–1082 (2011). 22020067 10.1038/nmeth.1742 PMC3431185

[b17] Kenthirapalan S., Waters A. P., Matuschewski K. & Kooij T. W. A. Flow cytometry-assisted rapid isolation of recombinant *Plasmodium berghei* parasites exemplified by functional analysis of aquaglyceroporin. Int. J. Parasitol. 42, 1185–1192 (2012). 23137753 10.1016/j.ijpara.2012.10.006 PMC3521960

[b18] Panatala R., Hennrich H. & Holthuis J. C. M. Inner workings and biological impact of phospholipid flippases. J. Cell Sci. 128, 2021–2032 (2015). 25918123 10.1242/jcs.102715

[b19] Marvin R. G. . Fluxes in ‘free' and total zinc are essential for progression of intraerythrocytic stages of *Plasmodium falciparum*. Chem. Biol. 19, 731–741 (2012). 22726687 10.1016/j.chembiol.2012.04.013 PMC3601789

[b20] Kenthirapalan S., Waters A. P., Matuschewski K. & Kooij T. W. A. Copper-transporting ATPase is important for malaria parasite fertility. Mol. Microbiol. 91, 315–325 (2014). 24237419 10.1111/mmi.12461 PMC4016742

[b21] Sahu T. . ZIPCO, a putative metal ion transporter, is crucial for *Plasmodium* liver-stage development. EMBO Mol. Med. 6, 1387–1397 (2014). 25257508 10.15252/emmm.201403868 PMC4237467

[b22] Portugal S. . Host-mediated regulation of superinfection in malaria. Nat. Med. 17, 732–737 (2011). 21572427 10.1038/nm.2368 PMC4200394

[b23] Mueller A.-K., Labaied M., Kappe S. H. I. & Matuschewski K. Genetically modified *Plasmodium* parasites as a protective experimental malaria vaccine. Nature 433, 164–167 (2005). 15580261 10.1038/nature03188

[b24] Butler N. S. . Superior antimalarial immunity after vaccination with late liver stage-arresting genetically attenuated parasites. Cell Host. Microbe 9, 451–462 (2011). 21669394 10.1016/j.chom.2011.05.008 PMC3117254

[b25] Haussig J. M., Matuschewski K. & Kooij T. W. A. Inactivation of a *Plasmodium* apicoplast protein attenuates formation of liver merozoites. Mol. Microbiol. 81, 1511–1525 (2011). 21848587 10.1111/j.1365-2958.2011.07787.x PMC3206223

[b26] Van Belle D. & André B. A genomic view of yeast membrane transporters. Curr. Opin. Cell Biol. 13, 389–398 (2001). 11454442 10.1016/s0955-0674(00)00226-x

[b27] Janse C. . High efficiency transfection of *Plasmodium berghei* facilitates novel selection procedures. Mol. Biochem. Parasitol. 145, 60–70 (2006). 16242190 10.1016/j.molbiopara.2005.09.007

[b28] Kooij T. W. A., Rauch M. M. & Matuschewski K. Expansion of experimental genetics approaches for *Plasmodium berghei* with versatile transfection vectors. Mol. Biochem. Parasitol. 185, 19–26 (2012). 22705315 10.1016/j.molbiopara.2012.06.001

[b29] Feldmann A. M. & Ponnudurai T. Selection of *Anopheles stephensi* for refractoriness and susceptibility to *Plasmodium falciparum*. Med. Vet. Entomol. 3, 41–52 (1989). 2519646 10.1111/j.1365-2915.1989.tb00473.x

[b30] Matz J. M., Matuschewski K. & Kooij T. W. A. Two putative protein export regulators promote *Plasmodium* blood stage development *in vivo*. Mol. Biochem. Parasitol. 191, 44–52 (2013). 24076174 10.1016/j.molbiopara.2013.09.003

[b31] Schindelin J. . Fiji: an open-source platform for biological-image analysis. Nat. Methods 9, 676–682 (2012). 22743772 10.1038/nmeth.2019 PMC3855844

[b32] Vanderberg J. P. Development of infectivity by the *Plasmodium berghei* sporozoite. J. Parasitol. 61, 43–50 (1975). 1090717

[b33] Friesen J. . Natural immunization against malaria: causal prophylaxis with antibiotics. Sci. Transl. Med. 2, 40ra49 (2010). 10.1126/scitranslmed.3001058 20630856

[b34] Nakabayashi H., Taketa K., Miyano K., Yamane T. & Sato J. Growth of human hepatoma cells lines with differentiated functions in chemically defined medium. Cancer Res. 42, 3858–3863 (1982). 6286115

[b35] Montagna G. N. . Antigen export during liver infection of the malaria parasite augments protective immunity. mBio 5, e01321 (2014). 25073641 10.1128/mBio.01321-14 PMC4128355

[b36] Tsuji M., Mattei D., Nussenzweig R. S., Eichinger D. & Zavala F. Demonstration of heat-shock protein 70 in the sporozoite stage of malaria parasites. Parasitol. Res. 80, 16–21 (1994). 8153120 10.1007/BF00932618

[b37] Matz J. M. & Kooij T. W. A. Towards genome-wide experimental genetics in the *in vivo* malaria model parasite *Plasmodium berghei*. Pathog. Glob. Health 109, 46–60 (2015). 25789828 10.1179/2047773215Y.0000000006 PMC4571819

[b38] Otto T. D. . A comprehensive evaluation of rodent malaria parasite genomes and gene expression. BMC Biol. 12, 86 (2014). 25359557 10.1186/s12915-014-0086-0 PMC4242472

[b39] Tarun A. S. . A combined transcriptome and proteome survey of malaria parasite liver stages. Proc. Natl Acad. Sci. USA 105, 305–310 (2008). 18172196 10.1073/pnas.0710780104 PMC2224207

[b40] Zhou Y. . Evidence-based annotation of the malaria parasite's genome using comparative expression profiling. PLoS ONE 3, e1570 (2008). 18270564 10.1371/journal.pone.0001570 PMC2215772

