# Supplementary material for: Functional profiles of orphan membrane transporters in the life cycle of the malaria parasite
Source: Nat Commun. 2016 Jan 22;7:10519. doi: 10.1038/ncomms10519 (PMC4736113; doi:10.1038/ncomms10519)
Supplement: Supplementary Information — Supplementary Figures 1-5, Supplementary Tables 1-3 and Supplementary References [file ncomms10519-s1.pdf]

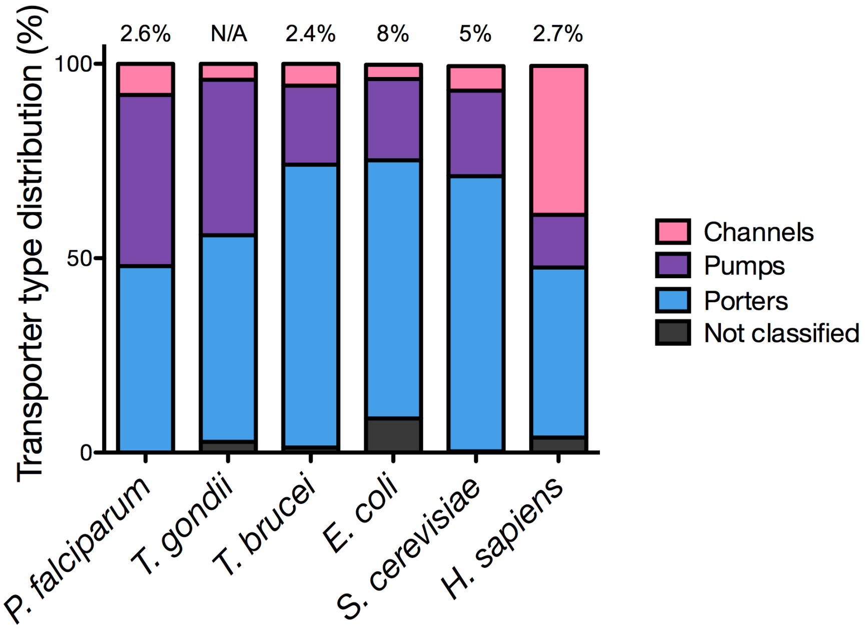

Supplementary Figure 1 | Comparison of membrane transport protein (MTP) type distribution in different organisms. Shown is the percentage of channels, pumps, and porters of the total MTPs in different organisms. The percentages on top of the bars indicate the proportion of all open reading frames in the respective genomes encoding MTPs (data from [http://www.membranetransport.org][1]).

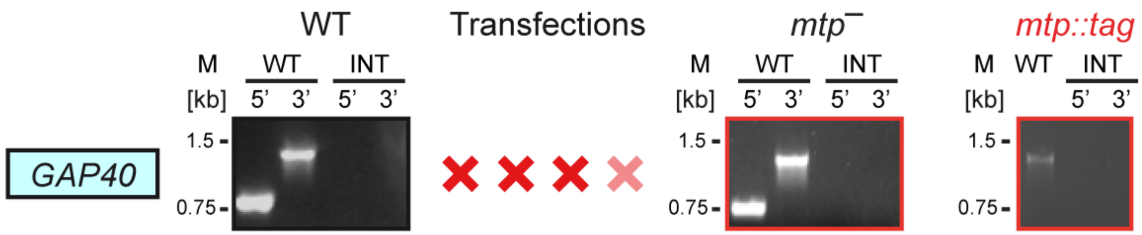

Supplementary Figure 2 | The glideosome associated protein 40 (*GAP40*) locus is inaccessible to genetic manipulation. Diagnostic PCR revealed absence of the desired gene replacement or endogenous tagging and presence of WT-specific PCR products (centre and right, red boxes) similar to those of the recipient parasites (left, black boxes; see Supplementary Fig. 5 for full gel pictures). Transfections were performed three times with the pMTP-KO vectors (red cross) and in the case of the gene deletion attempt once more with the respective *Plasmo*GEM vector (pink cross). Although inaccessibility of the *GAP40* locus to genetic manipulation cannot be formally excluded, it is likely essential in asexual blood stage parasites while endogenous tagging of the membrane-associated protein with the mCherry-3xMyc tag interferes with its function. In *Toxoplasma gondii*, GAP40 is associated with the glideosome[2].

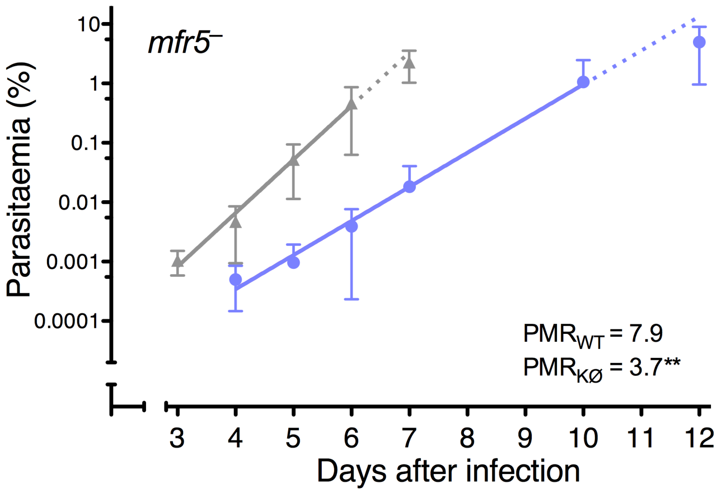

Supplementary Figure 3 | Individual blood infection of *mfr5⁻* parasites. Blood infection of the slowest growing parasite line, *mfr5⁻* (violet) compared with WT (grey) when grown in individual mice. Shown are mean parasitaemias ± S.D. from three experiments monitored by flow cytometry. Data from the exponential growth phase, *i.e.* with parasitemia <1%, fitted a linear regression well ($r^2 \geq 0.99$) and allowed the calculation of the parasite multiplication rate (PMR) from the slopes. **, p<0.01; (two-tailed F-test).

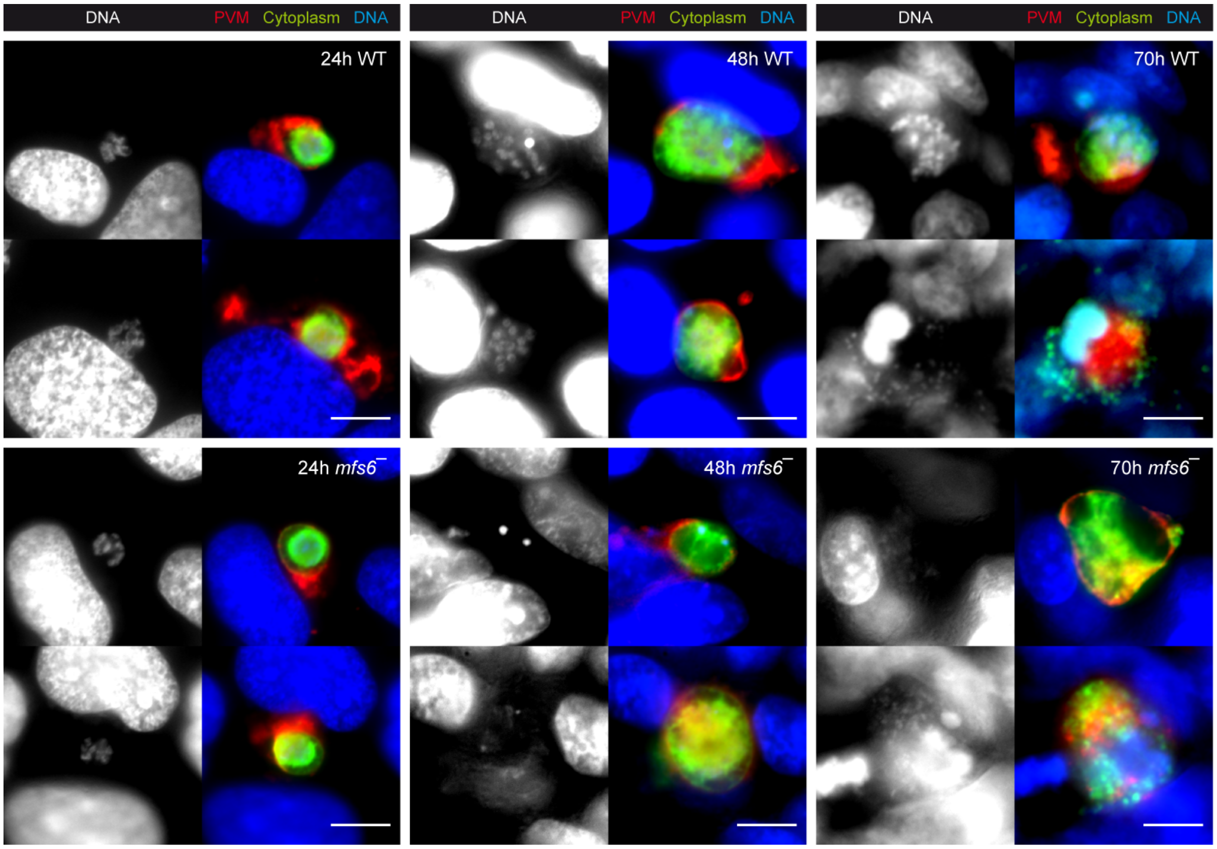

Supplementary Figure 4 | Aberrant liver-stage maturation of *mfs6⁻* parasites. Shown are two representative immunofluorescent micrographs each of cultured hepatoma cells at 24, 48, and 70 h after infection with WT or *mfs6⁻* salivary gland-associated sporozoites. (UIS4, parasite vacuole membrane, red; HSP70, cytoplasm, green; Hoechst, DNA, blue). Bars, 10 µm.

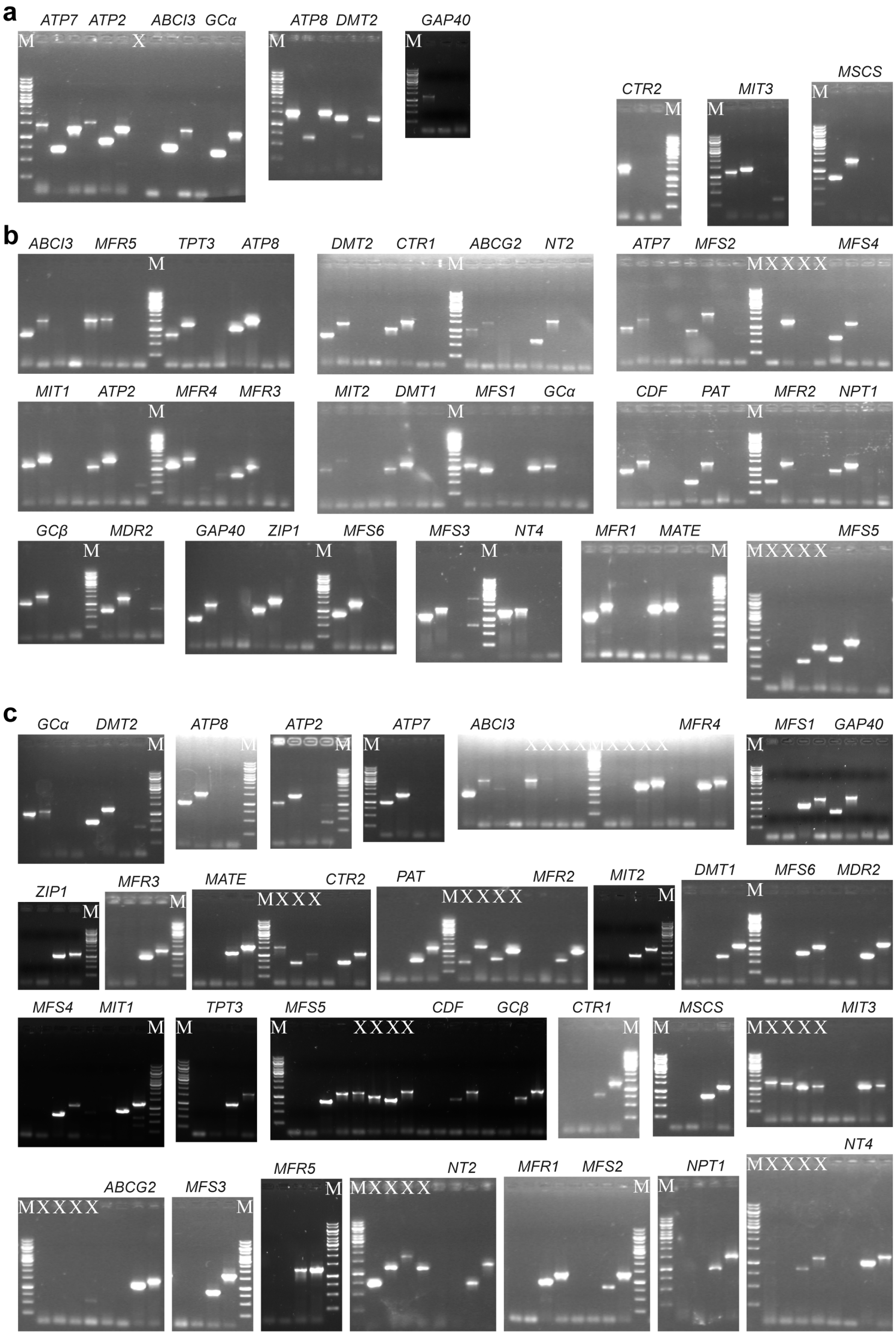

Supplementary Figure 5 | Full gel pictures. Diagnostic PCR of (**a**) parental populations with endogenously tagged genes, (**b**) WT parasites with the intact gene loci, and (**c**) parental and isogenic populations for essential and targetable genes, respectively, after transfection. M, lanes with 1kb DNA marker; X, lanes irrelevant to this study. The primers used from left to right are for 5’ and 3’ WT, 5’ and 3’ integration (note that for tagging genes and *CTR2* a single overarching WT PCR was performed).

Supplementary Table 1 | *Plasmodium berghei* genes encoding membrane transport proteins targeted in this study (Fig 1a).

Gene	*Pb* ID	*Pf* ID	Predicted product	Ref
**Channels/Pores – 1.A.23 The Small Conductance Mechanosensitive Ion Channel (MscS) Family**				
MSCS	PBANKA_093900	PF3D7_1107900	mechanosensitive ion channel protein	
**Channels/Pores – 1.A.35 The CorA Metal Ion Transporter (MIT) Family**				
MIT1	PBANKA_092790	PF3D7_1120300	metal ion channel - Mg^{2+}, Co^{2+} and Ni^{2+}	3
MIT2	PBANKA_140270	PF3D7_1304200	CorA-like Mg^{2+} transporter protein, putative	
MIT3	PBANKA_101700	PF3D7_1427600	CorA-like Mg^{2+} transporter protein, putative	
**Channels/Pores – 1.A.56 The Copper Transporter (Ctr) Family**				
CTR1	PBANKA_130290	PF3D7_1439000	copper transporter	4
CTR2	PBANKA_102150	PF3D7_1421900	Ctr copper transporter domain containing protein, putative	
**Primary Active Transporters/Pumps – 3.A.1: The ATP-binding Cassette (ABC) Superfamily**				
ABCG2	PBANKA_101810	PF3D7_1426500	ATP-binding cassette sub-family G member 2 (ABCG2)	5
ABCI3	PBANKA_121880	PF3D7_0319700	ABC transporter, putative	
MDR2	PBANKA_131170	PF3D7_1447900	multidrug resistance protein 2 (heavy metal transport family)	6
**Primary Active Transporters/Pumps – 3.A.3 The P-type ATPase (P-ATPase) Superfamily**				
ATP2	PBANKA_143480	PF3D7_1219600	aminophospholipid-transporting P-ATPase (ATPase2)	6
ATP7	PBANKA_080630	PF3D7_0319000	P-type ATPase, putative (ATPase7)	
ATP8	PBANKA_143830	PF3D7_1223400	phospholipid-transporting ATPase, putative (ATPase8)	
GCα	PBANKA_091030	PF3D7_1138400	aminophospholipid-transporting P-ATPase & guanylyl cyclase domains	7,8
GCβ	PBANKA_113670	PF3D7_1360500	aminophospholipid-transporting P-ATPase & guanylyl cyclase domains	9-11
	No *Pb* orthologue	PF3D7_1468600	aminophospholipid transporter, putative	
**Electrochemical Potential-driven Transporters – 2.A.1 The Major Facilitator Superfamily (MFS)**				
MFS1	PBANKA_123130	PF3D7_0516500	metabolite/drug transporter, putative	10,11
MFS2	PBANKA_081700	PF3D7_0916000	sugar transporter, putative	
MFS3	PBANKA_082040	PF3D7_0919500	sugar transporter, putative	
MFS4	PBANKA_060240	PF3D7_1203400	transporter, putative	
MFS5	PBANKA_101640	PF3D7_1428200	metabolite/drug transporter, putative	
MFS6	PBANKA_130470	PF3D7_1440800	major facilitator superfamily, putative	
PAT	PBANKA_030390	PF3D7_0206200	pantothenate transporter	12,13
**Electrochemical Potential-driven Transporters – The Major Facilitator Superfamily Related Transporters (MFR)**				
MFR1	PBANKA_011250	PF3D7_0614300	organic anion transporter	
MFR2	PBANKA_020840	PF3D7_0104700	transporter, putative	
MFR3	PBANKA_041050	PF3D7_0312500	transporter, putative	
MFR4	PBANKA_081570	PF3D7_0914700	transporter, putative	
MFR5	PBANKA_091830	PF3D7_1129900	transporter, putative	
NPT1	PBANKA_020830	PF3D7_0104800	novel putative transporter 1	14
	No *Pb* orthologue	PF3D7_0529200	sugar transporter, putative	
**Electrochemical Potential-driven Transporters – 2.A.4 The Cation Diffusion Facilitator (CDF) Family**				
CDF	PBANKA_142220	PF3D7_0715900	zinc transporter, putative	
**Electrochemical Potential-driven Transporters – 2.A.5 The Zinc (Zn^{2+})-Iron (Fe^{2+}) Permease (ZIP) Family**				
ZIP1	PBANKA_010770	PF3D7_0609100	Zn^{2+} or Fe^{2+} permease	
**Electrochemical Potential-driven Transporters – 2.A.7 The Drug/Metabolite Transporter (DMT) Superfamily**				
DMT1	PBANKA_142210	PF3D7_0715800	drug/metabolite exporter, drug/metabolite transporter	
DMT2	PBANKA_061460	PF3D7_0716900	drug metabolite transporter, putative	
GAP40	PBANKA_111530	PF3D7_0515700	glideosome-associated protein 40, putative	
TPT3	PBANKA_143400	PF3D7_1218400	triose or hexose phosphate/phosphate translocator, putative	3
**Electrochemical Potential-driven Transporters – 2.A.57 The Equilibrative Nucleoside Transporter (ENT) Family**				
NT2	PBANKA_070620	PF3D7_0824400	nucleoside transporter 2	15
	No *Pb* orthologue	PF3D7_1469400	nucleoside transporter 3, putative (NT3)	
NT4	PBANKA_020990	PF3D7_0103200	nucleoside transporter 4	16
**Electrochemical Potential-driven Transporters – 2.A.66.1 The Multi Antimicrobial Extrusion (MATE) Family**				
MATE	PBANKA_030970	PF3D7_0212800	multidrug efflux pump, putative	

Supplementary Table 2 | Details of the phenotyping screen (Fig. 1c and 3).

	Parasitemia (%)		Exflagellation (events/µl blood)		Sporozoites/ female mosquito		Transmission (days after infection)	
MSCS	10.2	6.1	12,300	nd	900	1,100	3	3
MIT1	9.4	6.1	16,000	nd	0	1,500	4	4
MIT2	15.3	19.0	18,600	20,000	0	0	-	-
MIT3	9.5	6.8	11,300	nd	3,500	7,500	4	4
CTR1	10.7	15.7	11,600	nd	0	700	-	-
CTR2	11.6	13.9	2,600	11,300	67,100	4,300	-	-
ABCG2	7.9	7.7	21,000	25,300	40,000	7,100	3	4
MDR2	11.0	7.1	15,300	nd	900	7,500	6	-
GCβ	6.2	5.7	8,600	nd	0	0	-	-
MFS1	9.1	9.5	11,300	11,400	0	500	-	-
MFS2	6.7	6.9	16,600	nd	0	0	-	-
MFS3	9.5	12.3	7,100	nd	500	1,600	4	5
MFS4	10.6	10.4	16,700	19,700	10,500	16,600	3	3
MFS5	7.9	6.8	6,300	nd	2,100	6,500	3	3
MFS6	4.1	4.5	3,700	4,700	12,100	20,000	-	-
PAT	6.3	7.4	2,800	1,500	0	0	-	-
MFR1	12.1	14.3	11,200	11,300	27,500	21,300	3	3
MFR2	4.4	3.5	5,000	nd	15,000	6,800	5	-
MFR3	9.5	9.0	6,000	nd	400	800	3	5
MFR4	12.8	9.5	15,300	nd	0	0	-	-
MFR5	0.9	1.3	0[a]	100[a]	0	0	-	-
NPT1	4.6	5.8	100	1,000	0	0	-	-
CDF	7.7	7.0	1,300	400	1,100	0	4	-
ZIP1	3.0	2.3	0	0	0	0	-	-
DMT1	6.9	3.5	21,800	nd	4,100	1,000	4	-
TPT3	6.3	9.2	15,100	nd	5,800	12,900	3	4
NT2	7.2	10.5	6,600	10,500	7,000	4,100	4	4
NT4	10.3	9.4	14,700	nd	0	0	-	-
MATE	11.8	12.8	27,300	nd	500	1,000	4	4
WT median	7.6		14,400		24,500		4	
n	22		16		14		14	
WT 10% percentile	4.6		5,900		2,500		- (n=1)[b]	
WT 25% percentile	6.1		6,800		6,900		5 (n=1)[b]	
WT 75% percentile	9.3		18,300		40,400		4 (n=7)[b]	
WT 90% percentile	10.8		25,300		50,100		3 (n=5)[b]	

[a] Due to slow asexual blood stage multiplication rate, the screen was continued by injecting $6x10^7$ *mfr5*⁻-infected erythrocytes instead of $1x10^7$, the inoculum for all other recombinant parasite lines. Parasitemias of the two mice, from which exflagellation was assessed, was 5.6 and 7.5%, respectively.
[b] Since mice remaining blood-film negative following infectious mosquito bites preclude the calculations of correct values for the percentile cut-offs, the counts of the prepatent periods of WT infections are indicated.

Supplementary Table 3 | Primer sequences and PCR product sizes.

Primer Name	Primer Sequence (restriction sites underlined)	WT[a]	INT[b]	Use[c]	Target	Ref
**Primers of general use**						
GFPrev	TGTGCCCATTAACATCACCATC			GT	GFP	17
mCherryRev	CCCTCCATGTGAACCTTGAAG			GT	mCherry	18
5’HSP70rev	CAATTTGTTGTACATAAAATAGGCAG			GT	5'PbHSP70	18
5’DHFRrev	ATGAAATACCGCTCCATTTTTCC			GT	5'PbDHFR-TS	18
pBAT-SQF	TATTTGCCTAACTATATTCAGGGG			SQ	PbSIL6L	
pBAT-SQR	TTATTTGTGCCTGAATTATAGTGC			SQ	PbSIL6R	
T7	TAATACGACTCACTATAGGG			GT	pBSKS	
***MSCS* – PBANKA_093900 – PF3D7_1107900 – mechanosensitive ion channel protein**						
5'MSCS-F-SacII	TTTCCGCGGCCATTATGTGCGTCTGAATCC	0.5		TV	5'PbMSCS	
5'MSCS-R-HpaI	TTTCAGCTGTTAATTAAGCGCATAATCCGTTTATTTACC			TV	5'PbMSCS	
3'MSCS-F-AvrII	AATCCTAGGTTCTCAAAATAAAAGATTTTCAGTGG	0.5		TV	3'PbMSCS	
3'MSCS-R-KpnI	ATAGGTACCAGTCATAACAATGTGGTAGTTCACC			TV	3'PbMSCS	
5'MSCS-F	AAGAGAGAAAGCTGGATAATGC	0.8	0.8	GT	5'PbMSCS	
5'MSCS-R	TGATTGTGGATACTTTGTCTTCG			GT	5'PbMSCS	
3'MSCS-F	TGGACAAAAATGAAAATGGGTTGG	1.6		GT	3'PbMSCS	
3'MSCS-R	TGGCTAAAAGCTTGTGAAATGG		1.4	GT	3'PbMSCS	
***MIT1* – PBANKA_092790 – PF3D7_1120300 – metal ion channel - Mg^{2+}, Co^{2+} and Ni^{2+}**						
5'MIT1-F-SacII	TTTCCGCGGCATATAGATTCACAAACTCATACCG	0.6		TV	5'PbMIT1	
5'MIT1-R-HpaI	TTTCAGCTGTTAATTAATATTATATATAACACAATAATATATTTTATAGTAAAAATTTATCG			TV	5'PbMIT1	
3'MIT1-F-AvrII	AATCCTAGGAGCATGTGAATATCACCAATATGC	0.5		TV	3'PbMIT1	
3'MIT1-R-KpnI	ATAGGTACCCCACTGTTATCAAGACAGAATTGC			TV	3'PbMIT1	
5'MIT1-F	CTCACCTTAAGCAAATATACTTGG	1.3	0.9	GT	5'PbMIT1	
5'MIT1-R	CAAATAGCCAAGTTTCATTATATCG			GT	5'PbMIT1	
3'MIT1-F	AGGAATTGTACAGATAATGGAAAGG	1.6		GT	3'PbMIT1	
3'MIT1-R	GATATATAAATTCATATTCACAATTTGATGG		1.4	GT	3'PbMIT1	
***MIT2* – PBANKA_140270 – PF3D7_1304200 – CorA-like Mg^{2+} transporter protein, putative**						
5'MIT2-F-SacII	TTTCCGCGGATTAATCGTTGGTGTTATTACATATGC	0.6		TV	5'PbMIT2	
5'MIT2-R-HpaI	TTTGTTAACTTAATTAAAAAAATTATCAAAGGGATGACGAG			TV	5'PbMIT2	
3'MIT2-F-AvrII	AATCCTAGGCGCTAAGTCACGAATAAAATGG	0.5		TV	3'PbMIT2	
3'MIT2-R-KpnI	ATAGGTACCCAAAATTAATAGAAAATGAGAAACACG			TV	3'PbMIT2	
5'MIT2-F	AATATTTGAATTCCTCCCAACTGC	1.1	1.1	GT	5'PbMIT2	
5'MIT2-R	TTATTCATAAATGTTGTGCTCATTCC			GT	5'PbMIT2	
3'MIT2-F	TATCAAATTTACATAACTTAAAAGAGCC	1.5		GT	3'PbMIT2	
3'MIT2-R	CACATCTCATTTTCACAGATGC		1.4	GT	3'PbMIT2	
***MIT3* – PBANKA_101700 – PF3D7_1427600 – CorA-like Mg^{2+} transporter protein, putative**						
5'MIT3-F-SacII	TTTCCGCGGAATTTATGTTTTTTATACACGGAATTGC	0.6		TV	5'PbMIT3	
5'MIT3-R-HpaI	TTTGTTAACTTAATTAAATAATAGATCAATATATATAATATGCGAATGC			TV	5'PbMIT3	
3'MIT3-F-AvrII	AATCCTAGGGAATATATTTTTATGCAATATCCTTTCG	0.5		TV	3'PbMIT3	
3'MIT3-R-KpnI	ATAGGTACCAAAAATTGTTAAAAATGAACTTTGATGC			TV	3'PbMIT3	
5'MIT3-F	AACATTTTTATGGAAGATAACTGTGC	1.3	1.1	GT	5'PbMIT3	
5'MIT3-R	TGCACAATTTTTAGTAATTCATGGG			GT	5'PbMIT3	
3'MIT3-F	CCCACATTTACAAATAACCCTATACC	1.4		GT	3'PbMIT3	
3'MIT3-R	GCTGTAGATTGTGTTTTATTTTTTCG		1.3	GT	3'PbMIT3	
***CTR1* – PBANKA_130290 – PF3D7_1439000 – copper transporter**						
5'CTR1-F-SacII	TTTCCGCGGAAATAATCCATTTAAAGATAATAAAATCGC	0.5		TV	5'PbCTR1	
5'CTR1-R-HpaI	TTTGTTAACTTAATTAAGGTTTAGTTATTTAGTTTGTTATTGTTATAAG			TV	5'PbCTR1	
3'CTR1-F-AvrII	AATCCTAGGACAAGATATTATTCTATATGTTTGTACTCC	0.5		TV	3'PbCTR1	
3'CTR1-R-KpnI	ATAGGTACCATTATTCTCCTATTTAAAGATATGGTTCC			TV	3'PbCTR1	
5'CTR1-F	TGTTGTTGGCGTAAAAATGTGC	1.0	0.8	GT	5'PbCTR1	
5'CTR1-R	GAATTTTGTTTTTGCCTTCACTTCC			GT	5'PbCTR1	
3'CTR1-F	GCCTATGTCATTTCAGAATACTACC	1.4		GT	3'PbCTR1	
3'CTR1-R	TATACGAAGCGACAATAAAATGC		1.5	GT	3'PbCTR1	
***CTR2* – PBANKA_102150 – PF3D7_1421900 – Ctr copper transporter domain containing protein, putative**						
5'CTR2-F-SacII	TTTCCGCGGTTCTTGTTAAATCCATTAAATTTTATTATCG	0.5		TV	5'PbCTR2	
5'CTR2-R-HpaI	TTTGTTAACTTAATTAAATTAGAAATTTTTGTATATCTTAAATTATCATTTG			TV	5'PbCTR2	
3'CTR2-F-AvrII	AATCCTAGGTAACAACTGTACTCAACATATTTTAAGC	0.5		TV	3'PbCTR2	
3'CTR2-R-KpnI	ATAGGTACCATATATACATAATATAATGGACAGATTATTAACC			TV	3'PbCTR2	
5'CTR2-F	TTTAACAATGTTGCACAATATAGTGG	1.6[d]	0.7	GT	5'PbCTR2	
3'CTR2-R	TCAAATAAGATGGTAGCTAAAATAGG		1.3	GT	3'PbCTR2	

Supplementary Table 3 | Primer sequences and PCR product sizes (continued…)

Primer Name	Primer Sequence (restriction sites underlined)	WT[a]	INT[b]	Use[c]	Target	Ref
***ABCG2* – PBANKA_101810 – PF3D7_1426500 – ATP-binding cassette sub-family G member 2 (ABCG2)**						
5'ABCG2-F-SacII	TTTCCGCGGTGAACATGCGGAAAAAGTATGC	0.5		TV	5'PbABCG2	
5'ABCG2-R-HpaI	TTTGTTAACTTAATTAAACGTGGAAAAATATGGGAAATGAG			TV	5'PbABCG2	
3'ABCG2-F-AvrII	AATCCTAGGATATATTATAATTAAAAATGTTATTTGCCTATCC	0.5		TV	3'PbABCG2	
3'ABCG2-R-KpnI	ATAGGTACCAAATATTTACGAGCTAGCTTAAATTGG			TV	3'PbABCG2	
5'ABCG2-F	GAAGTGAACGAAAATGAATATAAGC	1.1	1.2	GT	5'PbABCG2	
5'ABCG2-R	TGATGTCAAATCCTTTTTATTTTTATGC			GT	5'PbABCG2	
3'ABCG2-F	ACATCGGGTTTTAGATTCAATGG	1.5		GT	3'PbABCG2	
3'ABCG2-R	TTCTATTTTACGTGGTTTGACTTACC		1.3	GT	3'PbABCG2	
***ABCI3* – PBANKA_121880 – PF3D7_0319700 – ABC transporter, putative**						
5'ABCI3-F-SacII	TTTCCGCGGAAAAATAACAAATAGAAAAGATAAAGAATACG	0.5		TV	5'PbABCI3	
5'ABCI3-R-HpaI	TTTGTTAACTTAATTAAGAGTTTATTTTAAAAATATAATTAATTTTATTAGCAAAG			TV	5'PbABCI3	
CT-ABCI3-F-SacII	AATCCGCGGTTAAATTGATTGATAATAACTTTTCTTATGTTC	0.5		TV	CT-PbABCI3	
CT-ABCI3-R-HpaI	AAAGTTAACCGTCTTCAAAATCAATAGGAAAATATC			TV	CT-PbABCI3	
3'ABCI3-F-AvrII	AATCCTAGGTTCCAAAAACGTGGAAAAATAATAACC	0.5		TV	3'PbABCI3	
3'ABCI3-R-KpnI	ATAGGTACCCTTATGTACAGTCAATTACAAACAAGG			TV	3'PbABCI3	
5'ABCI3-F	TGAAAATATCAGTCGTTCTATAAGC	0.8	0.9	GT	5'PbABCI3	
5'ABCI3-R	CTTCTTTATTAAGCCATATAGTTTACG			GT	5'PbABCI3	
CT-ABCI3-F	as 3'ABCI3-F	1.5	0.8	GT	CT-PbABCI3	
3'ABCI3-F	GATTTTGAAATTCCTTGAAAAGTATGC	1.5		GT	3'PbABCI3	
3'ABCI3-R	TTGTTTTAAAGAATAATTTTGAGGAGC		1.5	GT	3'PbABCI3	
***MDR2* – PBANKA_131170 – PF3D7_1447900 – multidrug resistance protein 2 (heavy metal transport family) (MDR2)**						
5'MDR2-F-SacII	TTTCCGCGGAAAAATAGGATTAAAAAATATTACTTAACTGTATATATATATAG	n/a		TV	5'PbMDR2	
5'MDR2-R-HpaI	TTTGTTAACTTAATTAAATGTAATGCCTCTTTGTAAAAATAAACC			TV	5'PbMDR2	
3'MDR2-F-AvrII	AATCCTAGGTTTGGAAAGTAATCCAAATACTAATCG	0.5		TV	3'PbMDR2	
3'MDR2-R-KpnI	ATAGGTACCATAATTGCTTTATTTCATATATCGATGG			TV	3'PbMDR2	
5'MDR2-F	GTGCATATATTCGCCAAAACG	n/a	n/a	GT	5'PbMDR2	
5'MDR2-R	TCCGCAAAATGTTGCTATAAGG			GT	5'PbMDR2	
3'MDR2-F	TATATCGTTTTTATGATGCTGAAGG	1.4		GT	3'PbMDR2	
3'MDR2-R	CAAAAAATCTGGATATATTATTTATACATGC		1.5	GT	3'PbMDR2	
***ATP2* – PBANKA_143480 – PF3D7_1219600 – P-type ATPase, putative (ATPase2)**						
5'ATP2-F-SacII	TTTCCGCGGCCGGCGTACTAATATAACATTCC	0.6		TV	5'PbATP2	
5'ATP2-R-HpaI	TTTGTTAACTTAATTAAAAGCAGTGATATTATTACCACCTG			TV	5'PbATP2	
CT-ATP2-F-SacII	AATCCGCGGCCAAGTCCCTTCGTTTTTGG	0.5		TV	CT-PbATP2	
CT-ATP2-R-HpaI	AAAGTTAACTATAAGTTTATCCTGTTTTCTTATGAAATG			TV	CT-PbATP2	
3'ATP2-F-AvrII	AATCCTAGGTTCATTTTAGCATTGAAGAAATATGG	0.5		TV	3'PbATP2	
3'ATP2-R-KpnI	ATAGGTACCGGTAATGGCTCTAACCTTTCG			TV	3'PbATP2	
5'ATP2-F	TGCATTTAACATATTCTCCAATTAGC	1.2	1.1	GT	5'PbATP2	
5'ATP2-R	GAAGTAATCACTGAATTTGTATGGC			GT	5'PbATP2	
CT-ATP2-F	as 3'ATP2-F	1.6	0.9	GT	CT-PbATP2	
3'ATP2-F	ATGGGTTTTAAATAGTCTTTTTCACG	1.6		GT	3'PbATP2	
3'ATP2-R	ATTTTCCCTATTTATATGCACTTCG		1.4	GT	3'PbATP2	
***ATP7* – PBANKA_080630– PF3D7_0319000 – aminophospholipid-transporting P-ATPase (ATPase7)**						
5'ATP7-F-SacII	TTTCCGCGGCATTTAGATAATTCTAATTTGCTTGC	0.4		TV	5'PbATP7	
5'ATP7-R-HpaI	TTTGTTAACTTAATTAATCTTTGCACACAATATACGCG			TV	5'PbATP7	
CT-ATP7-F-SacII	AATCCGCGGACCATGTTATTGCCGTTAATGC	0.4		TV	CT-PbATP7	
CT-ATP7-R-HpaI	AAAGTTAACTGTTTTCTTTTTCAACATAAGAAATGAATTTTC			TV	CT-PbATP7	
3'ATP7-F-AvrII	AATCCTAGGGCATACGCTCGTAGAAAATATC	0.5		TV	3'PbATP7	
3'ATP7-R-KpnI	ATAGGTACCCTACATATTTTGGCCATCCATCC			TV	3'PbATP7	
5'ATP7-F	TTTATCATTGCGTAGACTAAAACG	1.0	1.1	GT	5'PbATP7	
5'ATP7-R	ATAAATCCGGTTCTCATTATTTTTCC			GT	5'PbATP7	
CT-ATP7-F	as 3'ATP7-F	1.5	0.7	GT	CT-PbATP7	
3'ATP7-F	ACCATTCTATATTTCACAATGATCG	1.5		GT	3'PbATP7	
3'ATP7-R	GTAATCATGCTTTTCATTTACATTGC		1.5	GT	3'PbATP7	

Supplementary Table 3 | Primer sequences and PCR product sizes (continued…)

Primer Name	Primer Sequence (restriction sites underlined)	WT[a]	INT[b]	Use[c]	Target	Ref
***ATP8* – PBANKA_143830 – PF3D7_1223400 – phospholipid-transporting ATPase, putative**						
5'ATP8-F-SacII	TTTCCGCGGGAAATAAATGAGTAAGTAGTAATATCTCC	0.6		TV	5'PbATP8	
5'ATP8-R-HpaI	TTTGTTAACTTAATTAATTTTTTAATCAAATATTCGAATCCGTTC			TV	5'PbATP8	
CT-ATP8-F-SacII	AATCCGCGGTAGGGTACTGTTGTTATGCTAGG	0.5		TV	CT-PbATP8	
CT-ATP8-R-EcoRI	AAAGAATTCAGCTAGCTTAAAAAATTGTGATGG			TV	CT-PbATP8	
3'ATP8-F-AvrII	AATCCTAGGTTACACTTTTGTGTGTGAAATTATGC	0.5		TV	3'PbATP8	
3'ATP8-R-KpnI	ATAGGTACCGGAATACATACCATCATTTCGTGAA			TV	3'PbATP8	
5'ATP8-F	ATGCTAGTAATTTTCCTTTATTTATGC	1.0	0.9	GT	5'PbATP8	
5'ATP8-R	CAAAGGAGCAACAAATGTAAAAACATACCC			GT	5'PbATP8	
CT-ATP8-F	as 3'ATP8-F	1.5	0.7	GT	CT-PbATP8	
3'ATP8-F	CAACATACCAAGAAATGCAATGC	1.5		GT	3'PbATP8	
3'ATP8-R	GTAATAAGAGTTGGCAAAAGAAAACC		1.7	GT	3'PbATP8	
***GCα* – PBANKA_091030 – PF3D7_1138400 – guanylyl cyclase (GCalpha)**						
5'GCα-F-SacII	TTTCCGCGGTTGTCACAAATATATCACACACACC	0.5		TV	5'PbGCα	
5'GCα-R-HpaI	TTTGTTAACTTAATTAAAATTTACAAAAAGAAATAATCGAAGG			TV	5'PbGCα	
CT-GCα-F-SacII	AATCCGCGGGCCGCTTAAGATATGATTTATGG	0.4		TV	CT-PbGCα	
CT-GCα-R-HpaI	AAAGTTAACCAAAATTGAACTTGTGTCTTTGGAAT			TV	CT-PbGCα	
3'GCα-F-AvrII	AATCCTAGGTAAATATTTCATGTTTTCACATAATTGC	0.5		TV	3'PbGCα	
3'GCα-R-KpnI	ATAGGTACCTTCATTTGCTTAAATACGATAAGTCC			TV	3'PbGCα	
5'GCα-F	ATATTTTGCCGATTTCATATGTATCC	1.1	0.8	GT	5'PbGCα	
5'GCα-R	TGTTATTTTATTCGAAGGGAAGC			GT	5'PbGCα	
CT-GCα-F	ATCCAGTAGATGGAACAGAACG	1.4	0.7	GT	CT-PbGCα	
3'GCα-F	TGGAAAAAATTAATGTCTCAGAAACC	1.4		GT	3'PbGCα	
3'GCα-R	TCATCTTGTTTTAAGGATGCAAGG		1.4	GT	3'PbGCα	
***GCβ* – PBANKA_113670 – PF3D7_1360500 – guanylyl cyclase beta (GCbeta)**						
5'GCβ-F-SacII	TTTCCGCGGTTGTGGAAATAACATCATCAGTG	0.5		TV	5'PbGCβ	
5'GCβ-R-HpaI	TTTGTTAACTTAATTAATATGAAAGTGGGCAAACAAAAAGG			TV	5'PbGCβ	
3'GCβ-F-AvrII	AATCCTAGGTACAAATGTCTTGCAGAATGCAG	0.5		TV	3'PbGCβ	
3'GCβ-R-KpnI	ATAGGTACCCGCAACAAAGAATTCATAAAAATG			TV	3'PbGCβ	
5'GCβ-F	CAAATTTAAGCGGCGTATGC	1.1	1.0	GT	5'PbGCβ	
5'GCβ-R	GAATGCACATAGTTAACAATCGC			GT	5'PbGCβ	
3'GCβ-F	TTACACTATGGTTCGTGTGTTGG	1.6		GT	3'PbGCβ	
3'GCβ-R	AGCTTATGTTATTAAACGTATTTACG		1.4	GT	3'PbGCβ	
***MFS1* – PBANKA_123130 – PF3D7_0516500 – metabolite/drug transporter, putative**						
5'MFS1-F-SacII	TTTCCGCGGAAGGAATAAAGGAAAAGGTTTATGC	0.5		TV	5'PbMFS1	
5'MFS1-R-HpaI	TTTGTTAACTTAATTAAATTCTTAAATTGACCATTAACAAACAAG			TV	5'PbMFS1	
3'MFS1-F-AvrII	AATCCTAGGAATTTTGCTGTACTATTTCTTTTGC	0.5		TV	3'PbMFS1	
3'MFS1-R-KpnI	ATAGGTACCTGATTCGTGTATGCAATTATTTACG			TV	3'PbMFS1	
5'MFS1-F	CATATTTCTATAAACTATACAACATAGTGG	1.2	0.9	GT	5'PbMFS1	
5'MFS1-R	TATTTCGAACCATCATTTTGTGG			GT	5'PbMFS1	
3'MFS1-F	TACAATAATGGCTCCCTATTTGG/GTTGTAGATATGTTCAAGTTAACACC	1.0		GT	3'PbMFS1	
3'MFS1-R	TGAAGCATACGAAGAAAATTAAGC		1.4	GT	3'PbMFS1	
***MFS2* – PBANKA_081700 – PF3D7_0916000 – sugar transporter, putative**						
5'MFS2-F-SacII	TTTCCGCGGGGGACTAGAGACAACAGTTACC	0.5		TV	5'PbMFS2	
5'MFS2-R-HpaI	TTTGTTAACTTAATTAATAGAATCAGCTCAATTACAATGC			TV	5'PbMFS2	
3'MFS2-F-AvrII	AATCCTAGGTATACACACATTTATATGCTTTATTTTCC	0.5		TV	3'PbMFS2	
3'MFS2-R-KpnI	ATAGGTACCAGTATATAGAGCCAAATAATAGACG			TV	3'PbMFS2	
5'MFS2-F	GAGCACAAACACATAACAGTGC	0.9	0.8	GT	5'PbMFS2	
5'MFS2-R	TCTTTTTCAGTTTTATCCATGTTAGC			GT	5'PbMFS2	
3'MFS2-F	TCGGAAAAACTATAATAGATCAGC	1.9		GT	3'PbMFS2	
3'MFS2-R	CATTTCGTTTTAAATTTATTCACTTTGC		1.5	GT	3'PbMFS2	
***MFS3* – PBANKA_082040 – PF3D7_0919500 – sugar transporter, putative**						
5'MFS3-F-SacII	TTTCCGCGGGATATATTTGTTCTCTTTTTCTATCTCC	0.5		TV	5'PbMFS3	
5'MFS3-R-HpaI	TTTGTTAACTTAATTAAGTTGGTATATGTTGTAAAATTTGTTTCG			TV	5'PbMFS3	
3'MFS3-F-AvrII	AATCCTAGGCTCCTTATTTTTATGTGTAGACTTTATC	0.5		TV	3'PbMFS3	
3'MFS3-R-KpnI	ATAGGTACCTGAATTAAGTGCGCGAGTATG			TV	3'PbMFS3	
5'MFS3-F	CAGTCTTTGAGTAATAATCTAGCC	1.2	0.9	GT	5'PbMFS3	
5'MFS3-R	AAACAAACTATAACAATTGAGCATCC			GT	5'PbMFS3	
3'MFS3-F	TTGTATTTACTATAATAACTACTAAATTGGC	1.6		GT	3'PbMFS3	
3'MFS3-R	GTTTAAAACATCTAATATTATCACTAATCC		1.8	GT	3'PbMFS3	

Supplementary Table 3 | Primer sequences and PCR product sizes (continued…)						
**Primer Name**	**Primer Sequence (restriction sites underlined)**	**WT[a]**	**INT[b]**	**Use[c]**	**Target**	**Ref**
***MFS4* – PBANKA_060240 – PF3D7_1203400 – transporter, putative**						
5'MFS4-F-SacII	TTTCCGCGGACCATGCTGGTTTTTAATAAATTTGG	0.5		TV	5'PbMFS4	
5'MFS4-R-HpaI	TTTGTTAACTGTATATGATTCCTCGTATGTATGC			TV	5'PbMFS4	
3'MFS4-F-AvrII	AATCCTAGGGTATATACAAATATAGCATCGATCCC	0.5		TV	3'PbMFS4	
3'MFS4-R-KpnI	ATAGGTACCTAGAATTTTTCAATAATTAAATTGTATTTGC			TV	3'PbMFS4	
5'MFS4-F	ACTCTTAAAATAACCTATACATATACGC	0.7	0.7	GT	5'PbMFS4	
5'MFS4-R	GGATATACACACACAATGTTTATGTACC			GT	5'PbMFS4	
3'MFS4-F	AAATACGTTGTTTGGGCTTTATGC	1.3		GT	3'PbMFS4	
3'MFS4-R	CAGAATTTTTAGATCAGCTTCAGG		1.3	GT	3'PbMFS4	
***MFS5* – PBANKA_101640 – PF3D7_1428200 – metabolite/drug transporter, putative**						
5'MFS5-F-SacII	TTTCCGCGGGTCGTATTTTTAATGTTTTTCACAAATG	0.4		TV	5'PbMFS5	
5'MFS5-R-HpaI	TTTGTTAACTTTTCAATCCTGTATACATATTCTTCG			TV	5'PbMFS5	
3'MFS5-F-AvrII	AATCCTAGGCTGTATTTTAGCATATATTATTTTGTCG	0.5		TV	3'PbMFS5	
3'MFS5-R-KpnI	ATAGGTACCATCTTTAATTGGTATACCCGAAGC			TV	3'PbMFS5	
5'MFS5-F	ATAGTATATGTGCAATGATGAATTGG	0.7	0.8	GT	5'PbMFS5	
5'MFS5-R	TATCAATGTATAAAATATTGTACTATCCCC			GT	5'PbMFS5	
3'MFS5-F	GGAATGTTAAATGGAATACCAAAGC	1.4		GT	3'PbMFS5	
3'MFS5-R	ATTAAAAACCAATTTAGGACGATCG		1.3	GT	3'PbMFS5	
***MFS6* – PBANKA_130470 – PF3D7_1440800 – major facilitator superfamily, putative**						
5'MFS6-F-SacII	TTTCCGCGGAATTAACATGCCATAAAAATGTTCTC	0.6		TV	5'PbMFS6	
5'MFS6-R-HpaI	TTTGTTAACCTTTTATTAAGAATGACTCTCCACC			TV	5'PbMFS6	
3'MFS6-F-AvrII	AATCCTAGGCATTCATCGTGCTATTTTCTTCC	0.5		TV	3'PbMFS6	
3'MFS6-R-KpnI	ATAGGTACCACATATTTACGCCTTTTTAGAAGC			TV	3'PbMFS6	
5'MFS6-F	CTTATTGCATATATAACTAAGAATGTGG	0.9	0.8	GT	5'PbMFS6	
5'MFS6-R	GCTGTAAAAAGGACTCAAACAACG			GT	5'PbMFS6	
3'MFS6-F	CAAATACAAATTAGCCTTTTTGTTTCC	1.5		GT	3'PbMFS6	
3'MFS6-R	GTAATATTTTTGATGCTGATTATAATACCC		1.3	GT	3'PbMFS6	
***PAT* – PBANKA_030390 – PF3D7_0206200 – metabolite/drug transporter, putative**						
5'PAT-F-SacII	TTTCCGCGGCCTTTTCCCCGTTTTATGTAGC	0.4		TV	5'PbPAT	
5'PAT-R-HpaI	TTTGTTAACGAAATGAACACTTAGGGGTAATGG			TV	5'PbPAT	
3'PAT-F-AvrII	AATCCTAGGGGACAATAATGGATACTTTTAGATTCG	0.6		TV	3'PbPAT	
3'PAT-R-KpnI	ATAGGTACCTGGGCATAGATTCTTTGTTTAGC			TV	3'PbPAT	
5'PAT-F	GATTTATTTACAAGTATACTTTACATTACACC	0.6	0.6	GT	5'PbPAT	
5'PAT-R	ATTTTCTCCTCAATACATGGGACC			GT	5'PbPAT	
3'PAT-F	GGATTTACTGGTACAATTCCATGG	1.4		GT	3'PbPAT	
3'PAT-R	TTAACCTGCGAAAAGAAACTCG		1.4	GT	3'PbPAT	
***MFR1* – PBANKA_011250 – PF3D7_0614300 – organic anion transporter**						
5'MFR1-F-SacII	TTTCCGCGGTTCTATTTTAAAACAATTGAAGGGATGAC	0.6		TV	5'PbMFR1	
5'MFR1-R-HpaI	TTTGTTAACTTAATTAATAAATAAGTATATATTTATATGTTATATTTGCTTGC			TV	5'PbMFR1	
3'MFR1-F-AvrII	AATCCTAGGGCACATATTCTAGTATTTCATTATACGC	0.5		TV	3'PbMFR1	
3'MFR1-R-KpnI	ATAGGTACCGGCCCATTTTCATTCTGTTATTTCC			TV	3'PbMFR1	
5'MFR1-F	AAATACTAGGTTAATGAATTGTATATACCC	1.3	0.9	GT	5'PbMFR1	
5'MFR1-R	AAAGGGATCATTTTTATATGCTAAGG			GT	5'PbMFR1	
3'MFR1-F	TTTTTGGGATATACTGCTTTATTCG	1.7		GT	3'PbMFR1	
3'MFR1-R	CTTAAAATAGTTGAAACATTAATGTGGG		1.5	GT	3'PbMFR1	
***MFR2* – PBANKA_020840 – PF3D7_0104700 – transporter, putative**						
5'NPT1-F-SacII	TTTCCGCGGCGTGCGTATGCATTCTTTATAC	0.5		TV	5'PbNPT1	
5'NPT1-R-HpaI	TTTGTTAACAAAAATAATTTTCGTTGTAATTGTATGC			TV	5'PbNPT1	
3'NPT1-F-AvrII	AATCCTAGGGCAAATGCTGCATGTTATTTATAG	0.5		TV	3'PbNPT1	
3'NPT1-R-KpnI	ATAGGTACCAATATTTTTGTCCCCTGTTCATATC			TV	3'PbNPT1	
5'NPT1-F	TATATATGCATACTAATAGCTTTCG	1.0	1.1	GT	5'PbNPT1	
5'NPT1-R	TTTATTTCTTCTTTTGCATATTTCTCC			GT	5'PbNPT1	
3'NPT1-F	GGAAAAATCAGGAGAGGATTACG	1.3		GT	3'PbNPT1	
3'NPT1-R	CGAAAAATATTGTATACTACCAGAACC		1.7	GT	3'PbNPT1	
***MFR3* – PBANKA_041050 – PF3D7_0312500 – transporter, putative**						
5'MFR3-F-SacII	TTTCCGCGGCAATAAAATAGCAAATGGAAAAGAGG	0.5		TV	5'PbMFR3	
5'MFR3-R-HpaI	TTTGTTAACTCCTCCTATAATTCCCTTATAGCC			TV	5'PbMFR3	
3'MFR3-F-AvrII	AATCCTAGGGTATTTAAAACAGTTCAGAAACATTCCC	0.5		TV	3'PbMFR3	
3'MFR3-R-KpnI	ATAGGTACCTTATGTTTCTAAACAGAATTGAGCC			TV	3'PbMFR3	
5'MFR3-F	ATAATTTGTTAATATTGTAACTACAAAAAAGG	0.7	0.9	GT	5'PbMFR3	
5'MFR3-R	TTGCACATTTTCGTATAAATTCCG			GT	5'PbMFR3	
3'MFR3-F	AGAATTCTTTATTTTAACATTTAGTAATTGC	1.2		GT	3'PbMFR3	
3'MFR3-R	TTTTTCACTTTCTAAACTTACATATACG		1.3	GT	3'PbMFR3	

Supplementary Table 3 | Primer sequences and PCR product sizes (continued…)

Primer Name	Primer Sequence (restriction sites underlined)	WT[a]	INT[b]	Use[c]	Target	Ref
***MFR4* – PBANKA_081570 – PF3D7_0914700 – transporter, putative**						
5'MFR4-F-SacII	TTTCCGCGGGATAAAGTTTATTTAAAACATCATTGTGC	0.6		TV	5'PbMFR4	
5'MFR4-R-HpaI	TTTGTTAACTTAATTAATGAAATTAAAATATTTGGCTACTTTTGG			TV	5'PbMFR4	
3'MFR4-F-AvrII	AATCCTAGGGTAACTACGAATTATATGCACATCC	0.5		TV	3'PbMFR4	
3'MFR4-R-KpnI	ATAGGTACCAATTCTGAACATGTCATAAAAATAAAGG			TV	3'PbMFR4	
5'MFR4-F	GTATTTGTTGTATAATTGGGACATGC	1.3	1.1	GT	5'PbMFR4	
5'MFR4-R	AACTCGAAATGATTCTATTACATCG			GT	5'PbMFR4	
3'MFR4-F	ATAGGAAGTGAAATTGCTACTGG	1.7		GT	3'PbMFR4	
3'MFR4-R	TGTGTTTATGAAGATACACCTTGC		1.5	GT	3'PbMFR4	
***MFR5* – PBANKA_091830 – PF3D7_1129900 – transporter, putative**						
5'MFR5-F-SacII	TTTCCGCGGTGCAACAAAAAGTATAACAACATCG	0.6		TV	5'PbMFR5	
5'MFR5-R-HpaI	TTTGTTAACTTAATTAAGCTTAATATTCGATATTATTATTATATATGGTATTG			TV	5'PbMFR5	
3'MFR5-F-AvrII	AATCCTAGGTATGCCGTGATTTTGATTTATATGC	0.5		TV	3'PbMFR5	
3'MFR5-R-KpnI	ATAGGTACCCTCATTTCCTTATCATTTTTCAACTCC			TV	3'PbMFR5	
5'MFR5-F	TTAAAATAGGGCGAGATAATAAAGG	1.5	1.3	GT	5'PbMFR5	
5'MFR5-R	TGACCCAAATATAAAAATTGTTTGAACC			GT	5'PbMFR5	
3'MFR5-F	TTTCCCCAGATCTAGATATTATAGG	1.6		GT	3'PbMFR5	
3'MFR5-R	AATTCTTGATAACGTTCGAAAAGC		1.4	GT	3'PbMFR5	
***NPT1* – PBANKA_020830– PF3D7_0104800 – novel putative transporter 1 (NPT1)**						
5'MFR2-F-SacII	TTTCCGCGGAATGAATATGTTTTGCCACTTATTATATGC	0.5		TV	5'PbMFR2	
5'MFR2-R-HpaI	TTTGTTAACTTAATTAAATATTGCGAAAACCTTGGTATGG			TV	5'PbMFR2	
3'MFR2-F-AvrII	AATCCTAGGTGTAGTTCCAGATTATTTCAAATTGC	0.5		TV	3'PbMFR2	
3'MFR2-R-KpnI	ATAGGTACCCTATTCATAGGATCCTCCTTTTGG			TV	3'PbMFR2	
5'MFR2-F	CACAAAGTTTTAAGGAATATGAATGC	0.6	0.7	GT	5'PbMFR2	
5'MFR2-R	TGTACAATTTGATTCATTCATTTCG			GT	5'PbMFR2	
3'MFR2-F	AAATTCAAGTGGAAATAAAAATGATCC/ AGAGATGCAACCATCTTTAATCC	1.4		GT	3'PbMFR2	
3'MFR2-R	CATTTCTTCTGTCAGGAAAGTCC		1.3	GT	3'PbMFR2	
***CDF* – PBANKA_142220 – PF3D7_0715900 – zinc transporter, putative**						
5'CDF-F-SacII	TTTCCGCGGATTTTACATAGGAATATAACAAAAACAAGC	0.6		TV	5'PbCDF	
5'CDF-R-HpaI	TTTGTTAACTTAATTAATCTTATTTTTTAATACTTTTTCTAGTACCG			TV	5'PbCDF	
3'CDF-F-AvrII	AATCCTAGGTTGCCAAAAAGTTAGCTAACGC	0.5		TV	3'PbCDF	
3'CDF-R-KpnI	ATAGGTACCCAAATGGTAGAGTGCGAAAATCG			TV	3'PbCDF	
5'CDF-F	GCTGCCTATTCAAACTATACTACC	1.1	0.9	GT	5'PbCDF	
5'CDF-R	AAACAAAGCTCCTATAATTTCTGC			GT	5'PbCDF	
3'CDF-F	TCCACCTAATGATTTTAAAGTAGGC	1.6		GT	3'PbCDF	
3'CDF-R	TATCAGCTTCAAACAATAATTTATTGG		1.4	GT	3'PbCDF	
***ZIP1* – PBANKA_010770 – PF3D7_0609100 – Zn^{2+} or Fe^{2+} permease**						
5'ZIP1-F-SacII	TTTCCGCGGATAGATTTTTGAAAGGGAACAATATTAGCC	0.6		TV	5'PbZIP1	
5'ZIP1-R-HpaI	TTTGTTAACTTAATTAAAAAGAAATACTTAGGCCCCTATATTGC			TV	5'PbZIP1	
3'ZIP1-F-AvrII	AATCCTAGGGGCCATAATATATAAGCAAAGAACG	0.5		TV	3'PbZIP1	
3'ZIP1-R-KpnI	ATAGGTACCCAATAATTTTAGAAACCTTGTGGATTATGG			TV	3'PbZIP1	
5'ZIP1-F	GGTTTTGTTTAGAAATGAATAACATCG	1.1	1.1	GT	5'PbZIP1	
5'ZIP1-R	TCTTTCCAAATAATCCCAAAATATAAGG			GT	5'PbZIP1	
3'ZIP1-F	GGGATTGAAGGTAGATAAATAAAAGG	1.7		GT	3'PbZIP1	
3'ZIP1-R	TGAAAAAAAATTGCAAATACCTATTTGG		1.4	GT	3'PbZIP1	
***DMT1* – PBANKA_142210 – PF3D7_0715800 – drug/metabolite exporter, drug/metabolite transporter**						
5'DMT1-F-SacII	TTTCCGCGGTGTGTTCCTTGGTTTCATAACG	0.5		TV	5'PbDMT1	
5'DMT1-R-HpaI	TTTGTTAACTTAATTAAATTATGGGAAAGCAAAATAACTATACC			TV	5'PbDMT1	
3'DMT1-F-AvrII	AATCCTAGGCGGTTGAATGTATCTATTTGAGC	0.5		TV	3'PbDMT1	
3'DMT1-R-KpnI	ATAGGTACCGCTTAAAGAAAGAGCATGATGG			TV	3'PbDMT1	
5'DMT1-F	TCACTTGCTTGTTTTTGTTATATTCC	1.0	0.7	GT	5'PbDMT1	
5'DMT1-R	ATACAAAAGCATAGAAAACAAAAGC			GT	5'PbDMT1	
3'DMT1-F	TTTTTAGCTATATTGTTATCCTTTATTGG	1.3		GT	3'PbDMT1	
3'DMT1-R	GGAATTTTTATCAAATAATTAACATTTCCG		1.4	GT	3'PbDMT1	

Supplementary Table 3 | Primer sequences and PCR product sizes (continued…)

Primer Name	Primer Sequence (restriction sites underlined)	WT[a]	INT[b]	Use[c]	Target	Ref
***DMT2* – PBANKA_061460 – PF3D7_0716900 – drug metabolite transporter, putative**						
5'DMT2-F-SacII	TTTCCGCGGCGTAAATAATATGTTTCACATTTCTACC	0.6		TV	5'PbDMT2	
5'DMT2-R-HpaI	TTTGTTAACTTAATTAACTAAATATATTGAAATGTTATGCTGTTTTTC			TV	5'PbDMT2	
CT-DMT2-F-SacII	AATCCGCGGGAAAAATATATGCATAAAATGGATGGAC	0.5		TV	CT-PbDMT2	
CT-DMT2-R-HpaI	AAAGTTAACTACCTTTGGCTTAATTAAGATATCTG			TV	CT-PbDMT2	
3'DMT2-F-AvrII	AATCCTAGGCAGATCACCAGTTTAAACCAATTAAG	0.5		TV	3'PbDMT2	
3'DMT2-R-KpnI	ATAGGTACCACATACATAATGTCAAGCAAAACATAG			TV	3'PbDMT2	
5'DMT2-F	TACAACAATGTGGTATTATATATTCATCC	0.8	0.9	GT	5'PbDMT2	
5'DMT2-R	AACAACAATAATGTTGCTTCCACC			GT	5'PbDMT2	
CT-DMT2-F	as 3'DMT2-F	1.4	0.7	GT	CT-PbDMT2	
3'DMT2-F	CAAATACTTTAAAAGTCGAAATACATGG	1.4		GT	3'PbDMT2	
3'DMT2-R	GTATACTGAATATACATTTAAAACATCACC		1.4	GT	3'PbDMT2	
***GAP40* – PBANKA_111530 – PF3D7_0515700 – glideosome-associated protein 40, putative (GAP40)**						
5'GAP40-F-SacII	TTTCCGCGGGTGCAATATTTATTAACAATTAATATTATTAGC	0.5		TV	5'PbGAP40	
5'GAP40-R-HpaI	TTTGTTAACTTTTATTACTAAATTAAATAAATCTGTCTATGC			TV	5'PbGAP40	
3'GAP40-F-AvrII	AATCCTAGGGTGTATGTGTGCCTATCATTGC	0.5		TV	3'PbGAP40	
3'GAP40-R-KpnI	ATAGGTACCCGCAAACAGAATATCTATAAGTCC			TV	3'PbGAP40	
5'GAP40-F	CAATCTCAAAGTTACACACAATTCC	0.7	0.8	GT	5'PbGAP40	
5'GAP40-R	ACTCTGGCAAAATTGACTTTTCC			GT	5'PbGAP40	
3'GAP40-F	TTTATGATTGGTTTAGGATATAATAGACC	1.3		GT	3'PbGAP40	
3'GAP40-R	TTATGGGAATTCTTTTTCCTAATGC		1.4	GT	3'PbGAP40	
***TPT3* – PBANKA_143400 – PF3D7_1218400 – triose or hexose phosphate/phosphate translocator, putative**						
5'TPT3-F-SacII	TTTCCGCGGGATTTGTTCATTTATATTTCTTATCTTTTTAACC	0.6		TV	5'PbTPT3	
5'TPT3-R-HpaI	TTTGTTAACTTAATTAAATGTGTAGTCAAAAAAAATTAACAGATTGTC			TV	5'PbTPT3	
3'TPT3-F-AvrII	AATCCTAGGGTGTATATGTATGCCTTTTCATTTTCC	0.5		TV	3'PbTPT3	
3'TPT3-R-KpnI	ATAGGTACCGTCCCTCACGTGTATATGTGC			TV	3'PbTPT3	
5'TPT3-F	AAAGGAAGAACAAGTAGCAAATGG	0.8	1.0	GT	5'PbTPT3	
5'TPT3-R	TTGTTTGATCATCTTCATTTGTTATTTCATTTTGG			GT	5'PbTPT3	
3'TPT3-F	GATTTCCATTATTGTGGCTATATGC	1.3		GT	3'PbTPT3	
3'TPT3-R	TGGATCAAATACCTTACTGAATCG		1.8	GT	3'PbTPT3	
***NT2* – PBANKA_070620 – PF3D7_0824400 – nucleoside transporter 2 (NT2)**						
5'NT2-F-SacII	TTTCCGCGGAGCATGTATGTATAGTTATTTAAGCAG	0.5		TV	5'PbNT2	
5'NT2-R-HpaI	TTTGTTAACTTAATTAAACGAAAAAAAAATATATATATTATAAAAGGCTT			TV	5'PbNT2	
3'NT2-F-AvrII	AATCCTAGGATACATTTTTAATGCCTAAATTGAACG	0.4		TV	3'PbNT2	
3'NT2-R-KpnI	ATAGGTACCGCATGTGTGGTAATAAAATTATGATAGG			TV	3'PbNT2	
5'NT2-F	TTATTTTATTGGGAATAAGAGAATCTCC	0.7	0.8	GT	5'PbNT2	
5'NT2-R	CATTTGGAAATATGCTCGTGC			GT	5'PbNT2	
3'NT2-F	ATAAGTGGAAACTCAACTTCAGC	1.6		GT	3'PbNT2	
3'NT2-R	TTTGGAAAATTGTAAGAAATTGTTTATCC		1.6	GT	3'PbNT2	
***NT4* – PBANKA_020990 – PF3D7_0103200 – nucleoside transporter 4 (NT4)**						
5'NT4-F-SacII	TTTCCGCGGAAATTATTATTGATCCATCATGTTGTGG	0.5		TV	5'PbNT4	
5'NT4-R-HpaI	TTTGTTAACTTAATTAATCTGGGTATGTATATATTTTCCTTAGC			TV	5'PbNT4	
3'NT4-F-AvrII	AATCCTAGGTGTGGTCCAATTGTTTTATCTTTTCC	0.5		TV	3'PbNT4	
3'NT4-R-KpnI	ATAGGTACCTTTTTAACACTTATCATCGCTTGG			TV	3'PbNT4	
5'NT4-F	GATATCGCAATCTTTTGATTTATATGG	1.4	1.1	GT	5'PbNT4	
5'NT4-R	CAAATAATGCGCTAAAACTAATTCC			GT	5'PbNT4	
3'NT4-F	GTAAACCACTAAATGATGTAGAAAAGG	1.6		GT	3'PbNT4	
3'NT4-R	TTTCTTAAAAGGTAAACAAAATTACGC		1.5	GT	3'PbNT4	
***MATE* – PBANKA_030970 – PF3D7_0212800 – multidrug efflux pump, putative**						
5'MATE-F-SacII	TTTCCGCGGAAAAAATCCAAATTATATCATAAAAAGAAAGC	0.5		TV	5'PbMATE	
5'MATE-R-HpaI	TTTGTTAACTTAATTAATCATACTGGTATTTCTTCCAAGATTTC			TV	5'PbMATE	
3'MATE-F-AvrII	AATCCTAGGGACTCAAATTTCCCACTTTTTTATACG	0.5		TV	3'PbMATE	
3'MATE-R-KpnI	ATAGGTACCATTTACTAAATATATTATTATACTTTCATGGGG			TV	3'PbMATE	
5'MATE-F	GAACCTAAACCTAAGCAAATAGC	1.5	1.2	GT	5'PbMATE	
5'MATE-R	CCCTCAACTACAGAATTTAATAGC			GT	5'PbMATE	
3'MATE-F	AATTTAACATCTGTTGCAGTATTTATCC	1.7		GT	3'PbMATE	
3'MATE-R	GAACTTTTTGTTTACTAGCAACTGG		1.8	GT	3'PbMATE	

[a] Sizes of the PCR products of forward and reverse primers on WT gDNA; carboxy-terminal *mtp::tag* integration-specific primers combined with their respective reverse 3' gene-specific primers.
[b] Sizes of the respective integration-specific PCR products; forward 5' and carboxy-terminal gene-specific primers combined with 5'HSP70rev (*mtp*⁻ lines) or mCherryRev (*mtp::tag* lines) and reverse 3' gene-specific primers combined with 5'DHFRrev.
[c] TV, primers used for construction of Transfection Vectors; GT, primers used for GenoTyping; SQ, primers used for sequencing the targeting sequences.
[d] For WT-specific diagnostic PCR the 5'CTR2-F and 3'CTR2-R were combined to amplify the complete locus.

SUPPLEMMENTARY REFERENCES

1. Ren, Q., Chen, K. & Paulsen, I. T. TransportDB: a comprehensive database resource for cytoplasmic membrane transport systems and outer membrane channels. *Nucleic Acids Res.* **35,** D274–9 (2007).
2. Frénal, K. *et al.* Functional dissection of the apicomplexan glideosome molecular architecture. *Cell Host Microbe* **8,** 343–357 (2010).
3. Fonager, J. *et al.* Development of the *piggyBac* transposable system for *Plasmodium berghei* and its application for random mutagenesis in malaria parasites. *BMC Genomics* **12,** 155 (2011).
4. Choveaux, D. L., Przyborski, J. M. & Goldring, J. D. A *Plasmodium falciparum* copper-binding membrane protein with copper transport motifs. *Malar. J.* **11,** 397 (2012).
5. Tran, P. N. *et al.* A female gametocyte-specific ABC transporter plays a role in lipid metabolism in the malaria parasite. *Nat. Commun.* **5,** 4773 (2014).
6. Zalis, M. G., Wilson, C. M., Zhang, Y. & Wirth, D. F. Characterization of the *pfmdr2* gene for *Plasmodium falciparum*. *Mol. Biochem. Parasitol.* **62,** 83–92 (1993).
7. Trottein, F. & Cowman, A. F. Molecular cloning and sequence of two novel P-type adenosinetriphosphatases from *Plasmodium falciparum*. *Eur. J. Biochem.* **227,** 214–225 (1995).
8. Fonager, J. *et al.* Reduced CD36-dependent tissue sequestration of *Plasmodium*-infected erythrocytes is detrimental to malaria parasite growth *in vivo*. *J. Exp. Med.* **209,** 93–107 (2012).
9. Carucci, D. J. *et al.* Guanylyl cyclase activity associated with putative bifunctional integral membrane proteins in *Plasmodium falciparum*. *J. Biol. Chem.* **275,** 22147–22156 (2000).
10. Hirai, M., Arai, M., Kawai, S. & Matsuoka, H. *Pb*GCβ is essential for *Plasmodium* ookinete motility to invade midgut cell and for successful completion of parasite life cycle in mosquitoes. *J. Biochem.* **140,** 747–757 (2006).
11. Moon, R. W. *et al.* A cyclic GMP signalling module that regulates gliding motility in a malaria parasite. *PLoS Pathog.* **5,** e1000599 (2009).
12. Augagneur, Y. *et al.* Identification and functional analysis of the primary pantothenate transporter, *Pf*PAT, of the human malaria parasite *Plasmodium falciparum*. *J. Biol. Chem.* **288,** 20558–20567 (2013).
13. Hart, R. J., Lawres, L., Fritzen, E., Ben Mamoun, C. & Aly, A. S. I. *Plasmodium yoelii* vitamin B5 pantothenate transporter candidate is essential for parasite transmission to the mosquito. *Sci. Rep.* **4,** 5665 (2014).
14. Boisson, B. *et al.* The novel putative transporter NPT1 plays a critical role in early stages of *Plasmodium berghei* sexual development. *Mol. Microbiol.* **81,** 1343–1357 (2011).
15. Downie, M. J. *et al. Pf*NT2, a permease of the equilibrative nucleoside transporter family in the endoplasmic reticulum of *Plasmodium falciparum*. *J. Biol. Chem.* **285,** 20827–20833 (2010).
16. Frame, I. J., Merino, E. F., Schramm, V. L., Cassera, M. B. & Akabas, M. H. Malaria parasite type 4 equilibrative nucleoside transporters (ENT4) are purine transporters with distinct substrate specificity. *Biochem. J.* **446,** 179–190 (2012).
17. Haussig, J. M., Matuschewski, K. & Kooij, T. W. A. Experimental genetics of *Plasmodium berghei* NFU in the apicoplast iron-sulfur cluster biogenesis pathway. *PLoS ONE* **8,** e67269 (2013).
18. Kenthirapalan, S., Waters, A. P., Matuschewski, K. & Kooij, T. W. A. Flow cytometry-assisted rapid isolation of recombinant *Plasmodium berghei* parasites exemplified by functional analysis of aquaglyceroporin. *Int. J. Parasitol.* **42,** 1185–1192 (2012).
